# PRKCSH enhances colorectal cancer radioresistance via IRE1α/XBP1s-mediated DNA repair

**DOI:** 10.1038/s41419-025-07582-4

**Published:** 2025-04-06

**Authors:** Hui Shen, Jing Jin, Nanxi Yu, Tingting Liu, Yongzhan Nie, Zhijie Wan, Yuanyuan Chen, Kun Cao, Ying Xu, Yijuan Huang, Chao Feng, Ruixue Huang, Yanyong Yang, Fu Gao

**Affiliations:** 1 Department of Radiation Medicine, Faculty of Naval Medicine, Naval Medical University, Shanghai, China; 2 Department of Central Laboratory, Affiliated Hospital of Jiaxing University, Jiaxing, China; 3 State Key Laboratory of Holistic Integrative Management of Gastrointestinal Cancers and National Clinical Research Center for Digestive Diseases, Xijing Hospital of Digestive Diseases, Fourth Military Medical University, Xi’ an, China; 4 Key Laboratory of Radiological Protection and Nuclear Emergency, National Institute for Radiological Protection, Chinese Center for Disease Control and Prevention, Beijing, China; 5 Institutes for Translational Medicine, State Key Laboratory of Radiation Medicine and Protection, Medical College of Soochow University, Suzhou, China; 6 Department of Occupational and Environmental Health, Xiangya School of Public Health, Central South University, Hunan Changsha, China; 7 Shanghai Key Laboratory of Nautical Medicine and Translation of Drugs and Medical Devices, Shanghai, China

**Keywords:** Prognostic markers, Apoptosis, Prognostic markers, Rectal cancer

## Abstract

Neoadjuvant radiotherapy is the standard treatment for locally advanced rectal cancer, but resistance to this therapy remains a significant clinical challenge. Understanding the molecular mechanisms of radioresistance and developing strategies to enhance radiosensitivity are crucial for improving treatment outcomes. This study investigated the role of PRKCSH in colorectal cancer radioresistance and its underlying mechanisms. Our results demonstrate that PRKCSH is upregulated in colorectal cancer cells following ionizing radiation. Inhibiting PRKCSH sensitized these cells to radiation by reducing clonogenic survival, promoting apoptosis, and impairing DNA damage repair. Mechanistically, PRKCSH inhibition reduced p53 ubiquitination and degradation by activating the ER stress IRE1α/XBP1s pathway after radiation exposure, which enhanced DNA repair and contributed to radioresistance. In preclinical CRC models, PRKCSH depletion suppressed tumor growth and increased radiosensitivity. Similarly, in patient-derived organoid models, PRKCSH knockdown reduced organoid growth post-radiotherapy. In rectal cancer patients receiving neoadjuvant radiotherapy, higher PRKCSH expression in post-treatment samples correlated with reduced tumor regression. These findings suggest that targeting PRKCSH diminishes radioresistance by impairing DNA repair through the modulation of ER stress. Furthermore, PRKCSH expression may serve as a biomarker for evaluating radiotherapy efficacy and clinical outcomes in rectal cancer patients undergoing neoadjuvant therapy.

## Introduction

Between 2013 and 2017, the global incidence and mortality rates of colorectal cancer (CRC) remained high, with an incidence of 38.7 cases per 100,000 people and a mortality rate of 13.9 per 100,000 [1]. CRC ranks third among cancers worldwide [2], with an increasing incidence in younger patients and a gradually rising mortality rate [3]. Although neoadjuvant radiotherapy is the standard treatment for locally advanced rectal cancer [4], its efficacy is often limited by the radioresistance of cancer cells, significantly affecting patient survival outcomes. Tumor radioresistance primarily arises from enhanced DNA damage repair mechanisms, reduced apoptosis, and the ability to adapt to radiotherapy-induced stress responses [5–7]. Understanding the molecular mechanisms underlying CRC radioresistance is essential for developing strategies to enhance the efficacy of radiotherapy.

The unfolded protein response (UPR), a cellular mechanism activated during endoplasmic reticulum (ER) stress, has emerged as a critical pathway in regulating tumor progression and therapeutic resistance [8]. Among the UPR branches, the IRE1α/XBP1s signaling axis plays a central role in mediating cellular survival under stress and is frequently co-opted by tumors to evade stress-induced cell death [9, 10]. PRKCSH, a protein primarily localized in the ER [11], has been identified as a selective activator of the IRE1α/XBP1s pathway, promoting tumor growth and survival by enhancing ER stress adaptation [12]. Recent studies have shown that PRKCSH is overexpressed in various cancers and is associated with poor clinical outcomes [13, 14]. Notably, Shin et al. demonstrated that PRKCSH directly interacts with IRE1α to promote its autophosphorylation and oligomerization, selectively activating XBP1s splicing and downstream signaling, while minimally affecting other UPR branches such as PERK or ATF6 [12]. This underscores PRKCSH’s central role in modulating ER stress and its potential significance in cancer biology.

While ER stress has been extensively studied in the context of tumor biology, the role of PRKCSH in radiotherapy-induced stress remains poorly understood. Radiotherapy induces a multifaceted stress response, combining ER stress, oxidative stress, and DNA double-strand breaks (DSBs), which collectively determine therapeutic outcomes [6, 15]. We hypothesize that PRKCSH contributes to CRC radioresistance by selectively activating the IRE1α/XBP1s signaling axis, which in turn modulates DNA damage repair and apoptosis. Furthermore, PRKCSH’s overexpression in CRC may enhance tumor cells’ ability to repair radiation-induced DNA damage, thereby promoting radioresistance.

In this study, we systematically investigate the role of PRKCSH in CRC radioresistance and its underlying molecular mechanisms. Through in vitro and in vivo experiments, we demonstrate that PRKCSH plays a crucial role in modulating radiotherapy-induced stress responses, particularly by stabilizing p53 and promoting DNA repair. This research not only provides new insights into the molecular basis of CRC radioresistance but also highlights PRKCSH as a potential therapeutic target to overcome this clinical challenge.

## Results

### PRKCSH is upregulated in CRC samples and associated with radiation resistance

**Fig. 1:**
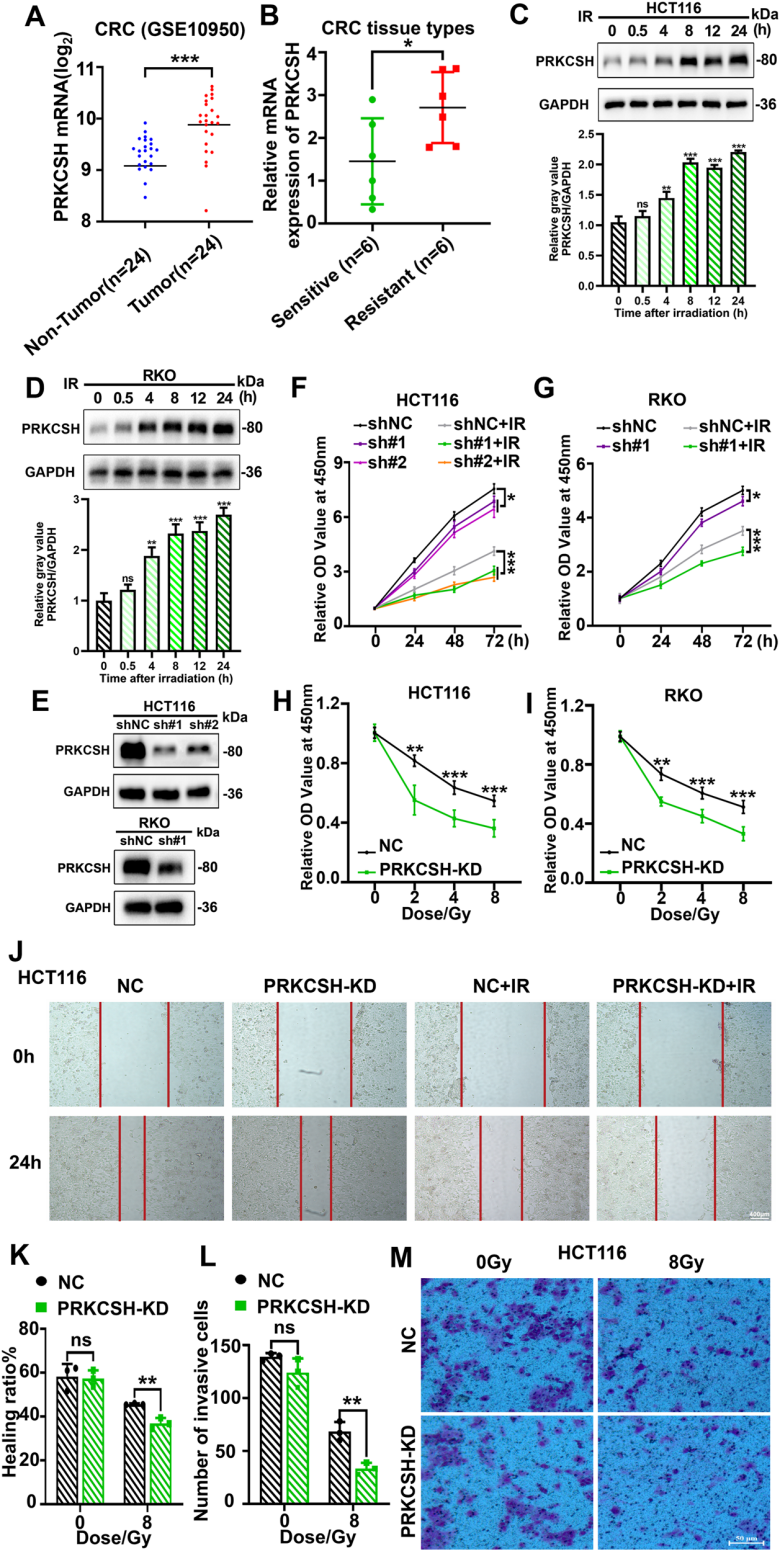
PRKCSH is upregulated in CRC samples and correlates with radioresistance. **A** PRKCSH mRNA levels in 24 human colorectal cancer (CRC) samples and matched normal tissues were analyzed using NCBI GEO microarray data. Results are expressed as mean ± SD and analyzed using a two-tailed Student’s *t*-test (***P < 0.001). **B** PRKCSH mRNA expression in radiation-resistant (*n* = 6) and radiation-sensitive (*n* = 6) CRC tissues was quantified. Data are presented as mean ± SD, with statistical analysis performed using a two-tailed Student’s *t*-test (*P < 0.05). **C** Western blot analysis confirmed PRKCSH expression levels at different time points post-8 Gy irradiation in HCT116 cells, with gray value analysis shown below. **D** Western blot analysis confirmed PRKCSH expression levels at different time points post-8 Gy irradiation in RKO cells, with gray value analysis shown below. **E** Western blot analysis confirmed PRKCSH knockdown efficiency in HCT116 and RKO CRC cell lines. **F**, **G** Cell proliferation in various CRC groups post-8 Gy irradiation was evaluated using the CCK-8 assay. **H**, **I** The CCK-8 assay was performed to evaluate cell proliferation across different radiation doses, including NC and PRKCSH-KD groups. Cell viability was measured at 72 h post-irradiation. **J**, **K** The scratch wound healing assay was used to assess the migration ability of HCT116 cells, including NC and PRKCSH-KD groups, at 24 h post-irradiation. **L**, **M** The Transwell invasion assay was conducted to evaluate the invasive capacity of HCT116 cells, including NC and PRKCSH-KD groups, at 24 h post-irradiation. Data represent mean ± SD from three independent experiments. Error bars indicate SD. Statistical significance: *P < 0.05, **P < 0.01, ***P < 0.001.

We analyzed PRKCSH expression in human colorectal cancer (CRC) using data from the GEO database, which revealed elevated levels in CRC tissues (Fig. 1A). To investigate the relationship between PRKCSH expression and radiotherapy outcomes, we examined tumor samples from 12 rectal cancer patients—six classified as resistant and six as sensitive to radiotherapy, as described in the Methods. PRKCSH mRNA levels were significantly higher in radiation-resistant tissues compared to radiation-sensitive ones (Fig. 1B), indicating a positive correlation with radiation resistance in CRC. To further validate the role of PRKCSH in radiation resistance, we analyzed the GSE226034 dataset (radiation-resistant group: *n* = 3; radiation-sensitive group: *n* = 3). The results indicated lower PRKCSH expression levels in the sensitive group, although one sample (GSM7061833) exhibited outlier behavior (Supplementary Fig. 2A). While the difference was not statistically significant, likely due to high individual variability in clinical samples and the small sample size, this trend was consistent with our experimental results, supporting the potential role of PRKCSH in radiation resistance.

Following 8 Gy irradiation, PRKCSH expression progressively increased within 24 h (Fig. 1C, D and Supplementary Fig. 1A, B) and then stabilized at later time points (48 and 72 h) (Supplementary Fig. 2B). This suggests that PRKCSH activation occurs early and remains sustained during the later stages. To evaluate its impact on cell viability and proliferation, we generated stable PRKCSH knockdown (PRKCSH-KD) and overexpression (PRKCSH-OE) cell lines (Fig. 1E, Supplementary Fig. 1C and Supplementary Fig. 2C). Viability was measured at 0, 24, 48, and 72 h post-irradiation, showing a significant decline in PRKCSH-KD HCT116, RKO and HT29 cells compared to controls (Fig. 1F, G and Supplementary Fig. 1D). As the radiation dose increased, proliferation decreased in both control and PRKCSH-KD groups, with PRKCSH-KD cells consistently exhibiting lower proliferation rates across all doses (Fig. 1H, I). Moreover, PRKCSH overexpression in HCT116 cells enhanced cell survival at different time points post-irradiation compared to control cells (Supplementary Fig. 2D). These findings underscore PRKCSH’s role in CRC radiation resistance.

Metastasis and invasion are key characteristics of malignancy and major contributors to cancer-related mortality. We assessed the impact of PRKCSH on HCT116 cell migration and invasion post-irradiation using wound healing and transwell assays. In the wound healing assay, significant closure occurred within 24 h in the control group, whereas the PRKCSH-KD group exhibited delayed healing (Fig. 1J, K). Similarly, in the invasion assay, PRKCSH knockdown significantly reduced the number of invasive cells, with fewer invasive cells observed in PRKCSH-KD post-irradiation compared to controls (Fig. 1L, M).

### Knockdown of PRKCSH increases radiosensitivity of CRC cells

**Fig. 2:**
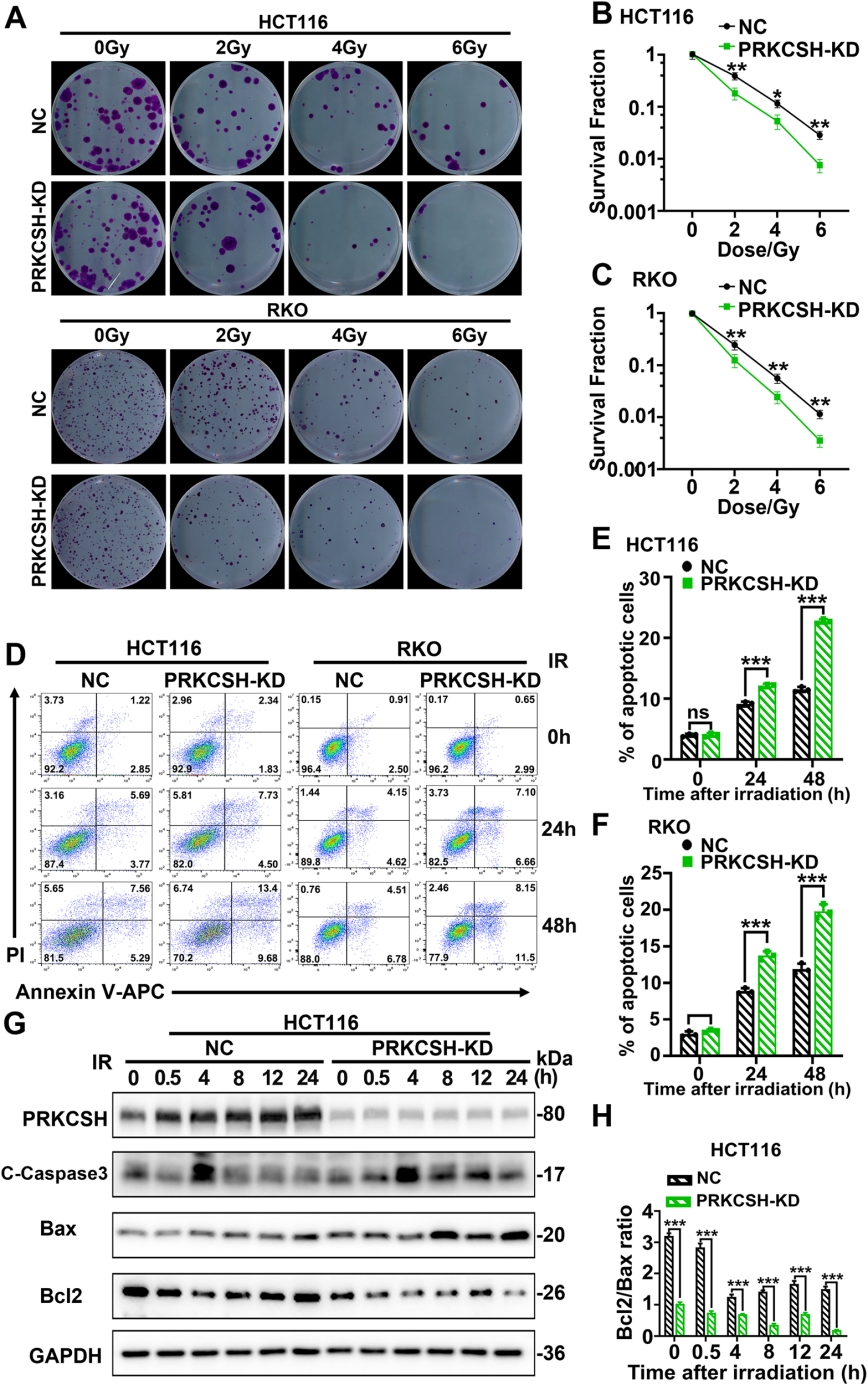
Knockdown of PRKCSH improves radiosensitivity of CRC cells. **A**–**C** Representative images and quantitative analysis of colony formation survival assays were performed on PRKCSH knockdown (PRKCSH-KD) and negative control (NC) cells following radiation at 0, 2, 4, and 6 Gy. **D**–**F** Apoptosis in colorectal cancer cells from different groups was assessed by flow cytometry at 24 and 48 h post-8 Gy radiation. In (**E**, **F**) apoptotic cells include both early and late apoptotic cells, as determined by Annexin V-APC positivity. **G** Western blot analysis detected the expression of apoptosis-related proteins in NC and PRKCSH-KD cells at various time points post-8 Gy radiation. **H** The relative Bcl2/Bax ratio was evaluated. Data are presented as mean ± standard deviation from three independent experiments. Error bars indicate standard deviation. Statistical significance is indicated as *P < 0.05, **P < 0.01, ***P < 0.001.

To evaluate the impact of PRKCSH on CRC cell radiosensitivity, we conducted a clonogenic survival assay. PRKCSH-knockdown HCT116, RKO and HT29 cells exhibited significantly lower clonogenic survival rates compared to controls across radiation doses ranging from 0 to 6 Gy (Fig. 2A–C and Supplementary Fig. 1E, F), indicating that PRKCSH knockdown enhances CRC cell radiosensitivity. Additionally, we compared radiation-induced apoptosis between PRKCSH-knockdown and control cells. Following 8 Gy irradiation, apoptotic cells were significantly more frequent in PRKCSH-knockdown HCT116, RKO and HT29 cells compared to controls (Fig. 2D–F and Supplementary Fig. 1G, H), whereas PRKCSH overexpression reduced apoptosis (Supplementary Fig. 2E).

After ionizing radiation, apoptosis-related protein expression was assessed in PRKCSH-knockdown (PRKCSH-KD) HCT116 cells, revealing higher Caspase-3 activation compared to controls. Silencing PRKCSH also increased Bax and decreased Bcl-2 levels post-radiation, resulting in a significantly lower Bcl-2/Bax ratio in the PRKCSH-KD group (Fig. 2G, H), suggesting that PRKCSH knockdown promotes apoptosis in CRC cells. Conversely, PRKCSH overexpression led to decreased Bax and increased Bcl-2 levels (Supplementary Fig. 2F, G), highlighting PRKCSH as a potential target for CRC radiotherapy. Overall, PRKCSH knockdown enhances CRC cell radiosensitivity.

### Knockdown of PRKCSH inhibits activation of IRE1α /XBP1s signaling pathway in radiation-induced ER stress

**Fig. 3:**
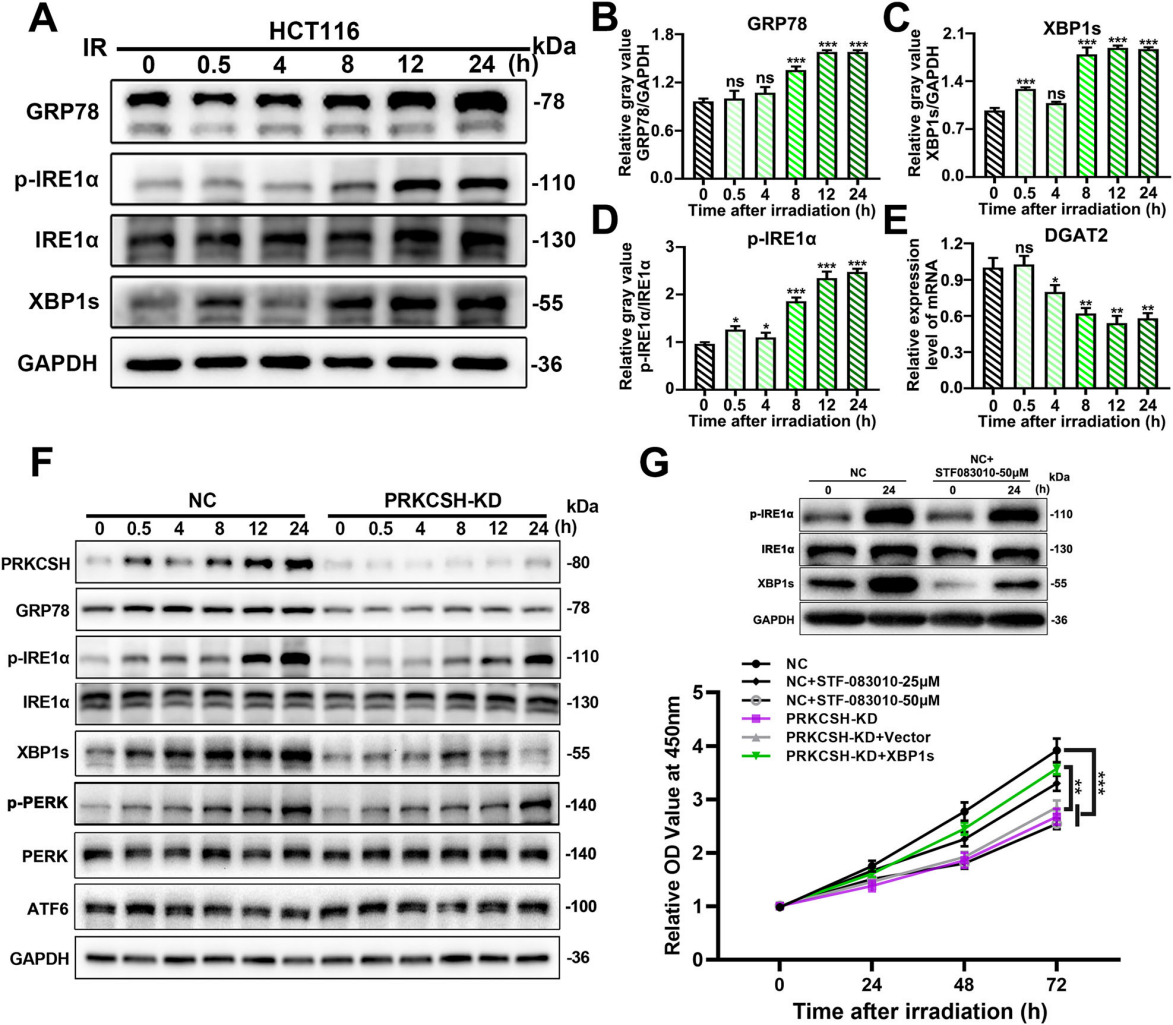
Knockdown of PRKCSH inhibits radiation-induced ER stress response. **A** Representative images of Western Blot of the protein expression in IRE1α/XBP1s pathway in HCT116 cells after 8 Gy IR at different time points. **B**, **D** Quantitative analysis of GRP78, p-IRE1α, IRE1α, and XBP1s protein levels. **E** Relative expression of DGAT2 mRNA in HCT116 cells at different time points post-irradiation. **F** Representative images of Western Blot of the protein expression in UPR pathway in NC and PRKCSH-KD cells after 8 Gy IR at different time points. **G** Relative OD values at 450 nm for HCT116 cells after 8 Gy irradiation, treated with NC, PRKCSH-KD, PRKCSH-KD + Vector, PRKCSH-KD + XBP1s, and the IRE1 inhibitor ATF-083010 (25 μM and 50 μM). Data are representative of three independent experiments with similar results. Error bars, SD. **P < 0.01, ***P < 0.001.

The IRE1α/XBP1s signaling pathway is a highly conserved mechanism activated in response to endoplasmic reticulum (ER) stress. Recent studies have shown that PRKCSH enhances IRE1α signaling activation during ER stress [12]. In our previous research, we observed a time-dependent increase in PRKCSH expression following ionizing radiation. To explore PRKCSH’s role in radiation-induced ER stress, we first confirmed that ionizing radiation activates ER stress through Western blot analysis. The results demonstrated a time-dependent increase in key ER stress markers, including GRP78, p-IRE1α, and XBP1s, post-8 Gy irradiation, confirming ER stress activation (Fig. 3A–D).

We further examined PRKCSH’s involvement in radiation-induced ER stress. PRKCSH-knockdown HCT116 cells exhibited significantly reduced levels of ER stress proteins (GRP78, p-IRE1α, and XBP1s) post-irradiation (Fig. 3F), while PRKCSH overexpression increased these levels (Supplementary Fig. 2F). Additionally, XBP1s overexpression restored the proliferation of PRKCSH-KD cells, reversing the inhibitory effect of PRKCSH knockdown on cell proliferation post-irradiation (Fig. 3G). XBP1s overexpression also rescued the clonogenic survival, invasion, and migration capabilities of PRKCSH-KD cells post-irradiation (Supplementary Fig. 3A–F). Furthermore, treatment with the specific IRE1 inhibitor STF-083010 [16] recapitulated the effects of PRKCSH knockdown, significantly enhancing radiosensitivity in HCT116 cells (Fig. 3G). These findings suggest that PRKCSH knockdown impairs the activation of the radiation-induced IRE1α/XBP1s pathway, thereby enhancing cellular radiosensitivity.

To assess the involvement of other UPR branches, we examined PERK activation following irradiation. Western blot analysis revealed no significant difference in total PERK levels between PRKCSH-knockdown and control groups (Fig. 3F). Given that PERK activation depends on phosphorylation, we measured p-PERK levels, which increased over time post-irradiation, confirming pathway activation. However, PRKCSH knockdown did not significantly affect p-PERK expression, indicating that PRKCSH predominantly regulates the IRE1α/XBP1s axis with minimal influence on the PERK pathway.

We also examined the activity of the RIDD pathway by monitoring DGAT2 mRNA levels, a well-established target of RIDD activity [17]. qPCR analysis revealed a significant decrease in DGAT2 expression post-irradiation, consistent with RIDD activation (Fig. 3E). This observation aligns with the expected function of RIDD in promoting selective mRNA degradation. However, as our primary focus is the IRE1α/XBP1s pathway, we did not further explore this aspect.

### PRKCSH deficiency inhibits DNA damage repair in CRC cells after IR

**Fig. 4:**
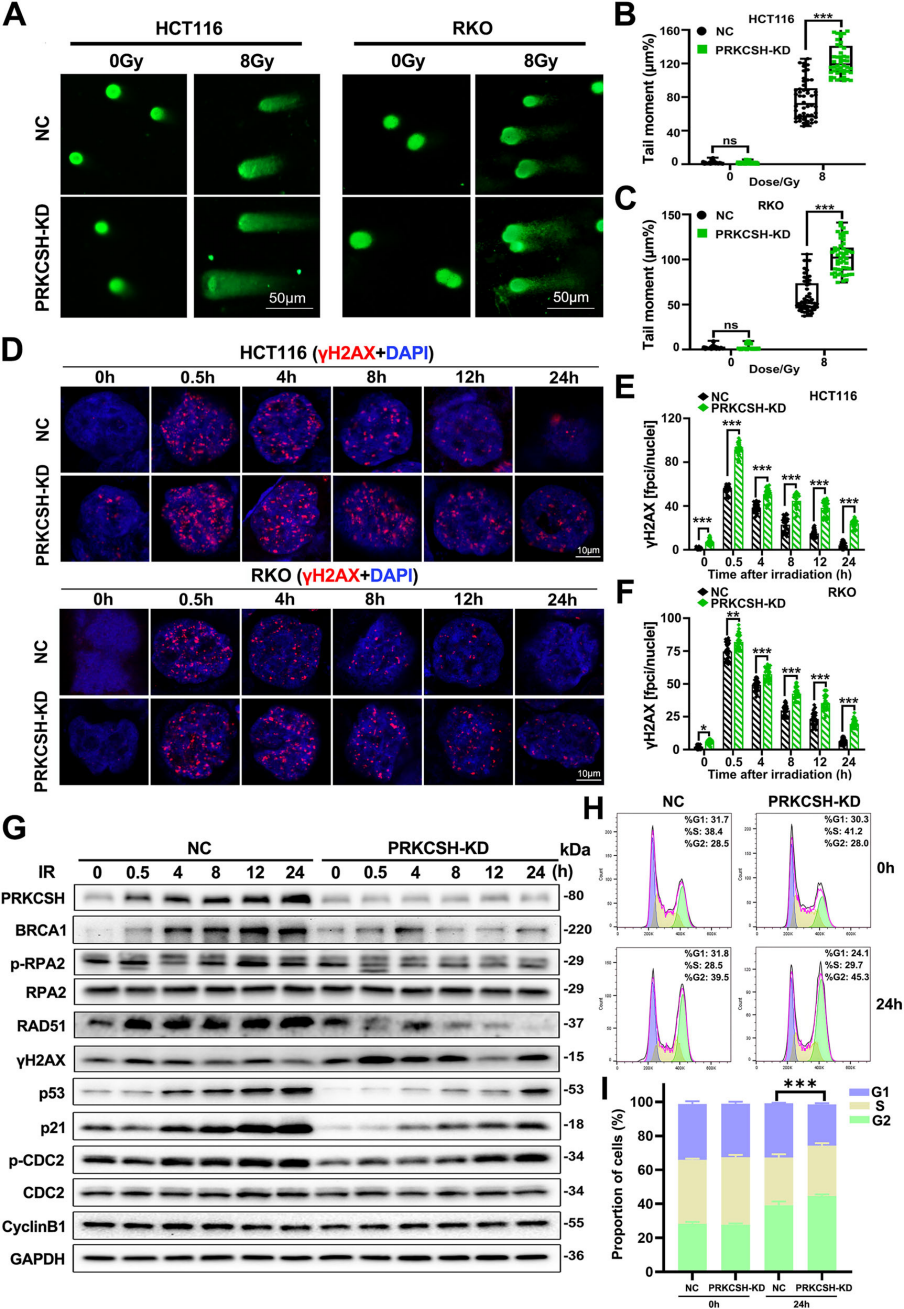
Knockdown of PRKCSH attenuates the radiation-induced DNA damage repair response. **A** Comet assay images depict the conditions of NC and KD cells 8 h after exposure to 8 Gy IR. **B**, **C** Tail moments in NC and KD cells were quantified using CASP 1.2.3b2 software. **D** Immunofluorescence staining was employed to detect γ-H2AX foci in HCT116 and RKO cells following 8 Gy IR. **E**, **F** The number of γ-H2AX foci per cell was quantified in HCT116 (**E**) and RKO (**F**) cells, with 30 cells per group at each time point. **G** Western blot images show the expression of DNA damage response and cell cycle proteins in HCT116-NC and HCT116-KD cells at various time points post-8 Gy IR. **H**, **I** The cell cycle distribution in HCT116 cells, treated with NC and PRKCSH-KD, was assessed at 0 and 24 h post-irradiation. Data are from three independent experiments and are presented with SD error bars. **P < 0.01, ***P < 0.001.

Radiotherapy induces tumor cell death by causing irreparable DNA double-strand breaks, leading to cell death [18]. We investigated the link between PRKCSH’s influence on CRC radiosensitivity and DNA damage repair mechanisms. Comet assay results showed that PRKCSH-knockdown HCT116 and RKO cells exhibited significantly higher tail moments compared to controls following 4 Gy irradiation, indicating increased DNA damage (Fig. 4A–C). In contrast, PRKCSH-overexpressing HCT116 cells had lower tail moments at the same dose (Supplementary Fig. 2H, I).

Following DNA double-strand breaks, γ-H2AX foci form as markers of DNA damage. We quantitatively analyzed γ-H2AX foci in individual cells using immunofluorescence staining. Microscopic images revealed that post-radiation, γ-H2AX foci formed at double-strand break sites and gradually decreased as DNA repair progressed. However, PRKCSH-silenced HCT116 and RKO cells retained significantly more γ-H2AX foci than controls, indicating impaired DNA repair (Fig. 4D–F). Further analysis showed that key DNA repair protein levels were suppressed in PRKCSH-knockdown cells (Fig. 4G).

The DNA damage repair process is closely linked to cell cycle regulation, with the cell cycle temporarily arresting to allow time for repair and ensure genomic stability. Our study found that PRKCSH knockdown weakened the G1 phase arrest following radiation (Fig. 4H, I), a finding further supported by the altered expression levels of cell cycle-related proteins (Fig. 4G). These results suggest that silencing PRKCSH impairs DNA damage repair after irradiation.

### PRKCSH promotes DNA damage repair through activation of the IRE1α/XBP1s pathway

**Fig. 5:**
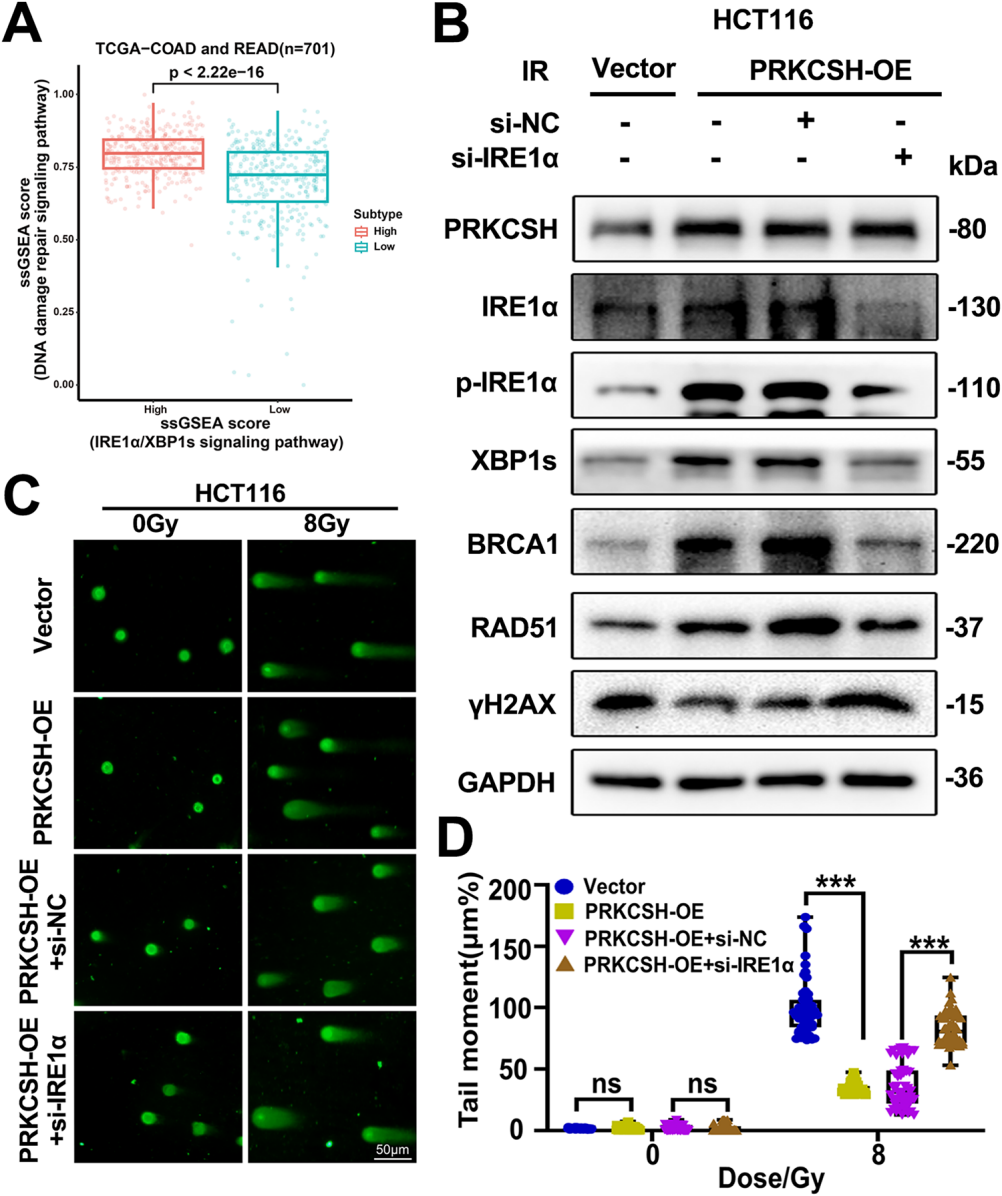
PRKCSH promotes DNA damage repair by activating the IRE1α/XBP1s pathway. **A** ssGSEA analysis reveals a significant positive correlation between the IRE1α/XBP1s signaling pathway and DNA damage repair. **B** Western blot analysis was performed to detect changes in key protein expression within the IRE1α/XBP1s and DDR pathways in Vector, PRKCSH-OE, PRKCSH-OE+si-NC, and PRKCSH-OE+si-IRE1α groups following ionizing radiation. **C** Comet assay was conducted to assess DNA damage in HCT116 cells transfected with Vector, PRKCSH-OE, PRKCSH-OE+si-NC, and PRKCSH-OE+si-IRE1α after ionizing radiation. **D** Tail moments in the different cell groups were quantified using CASP 1.2.3b2 software. Error bars represent SD. ns indicates no significant difference. **P < 0.01, ***P < 0.001.

Previous studies have demonstrated that the IRE1α/XBP1s pathway is activated during the DNA damage response, and IRE1α knockdown increases cellular sensitivity to ultraviolet radiation [19]. Consistently, our bioinformatics analysis further supports the functional link between the IRE1α/XBP1s pathway and DNA damage repair. By utilizing ssGSEA to quantify pathway activity scores from the TCGA-COAD and READ dataset (*n* = 701), we observed a significant positive correlation between the activation of the IRE1α/XBP1s pathway and DNA damage repair indicators. Notably, samples with high IRE1α/XBP1s pathway activity exhibited significantly elevated DNA damage repair scores compared to those with low pathway activity (Fig. 5A, *p* < 2.22e-16). This finding underscores the potential regulatory role of the IRE1α/XBP1s pathway in enhancing DNA damage repair capacity. We hypothesized that PRKCSH influences DNA damage repair through the IRE1α/XBP1s pathway, which is involved in the ER stress response. As shown in Fig. 5B, PRKCSH overexpression activated the IRE1α/XBP1s pathway and enhanced key DNA damage repair molecules. However, when IRE1α was silenced using siRNA, PRKCSH overexpression failed to activate downstream molecules such as XBP1s or key DNA repair components. Similarly, comet assay results showed that PRKCSH overexpression facilitated rapid radiation-induced DNA repair (measured by tail moment), but this repair was impaired when IRE1α was knocked down (Fig. 5C, D). These findings suggest that PRKCSH promotes DNA damage repair by activating the IRE1α/XBP1s pathway.

### PRKCSH inhibits ubiquitinated degradation of p53 via XBP1s to promote DNA damage repair

**Fig. 6:**
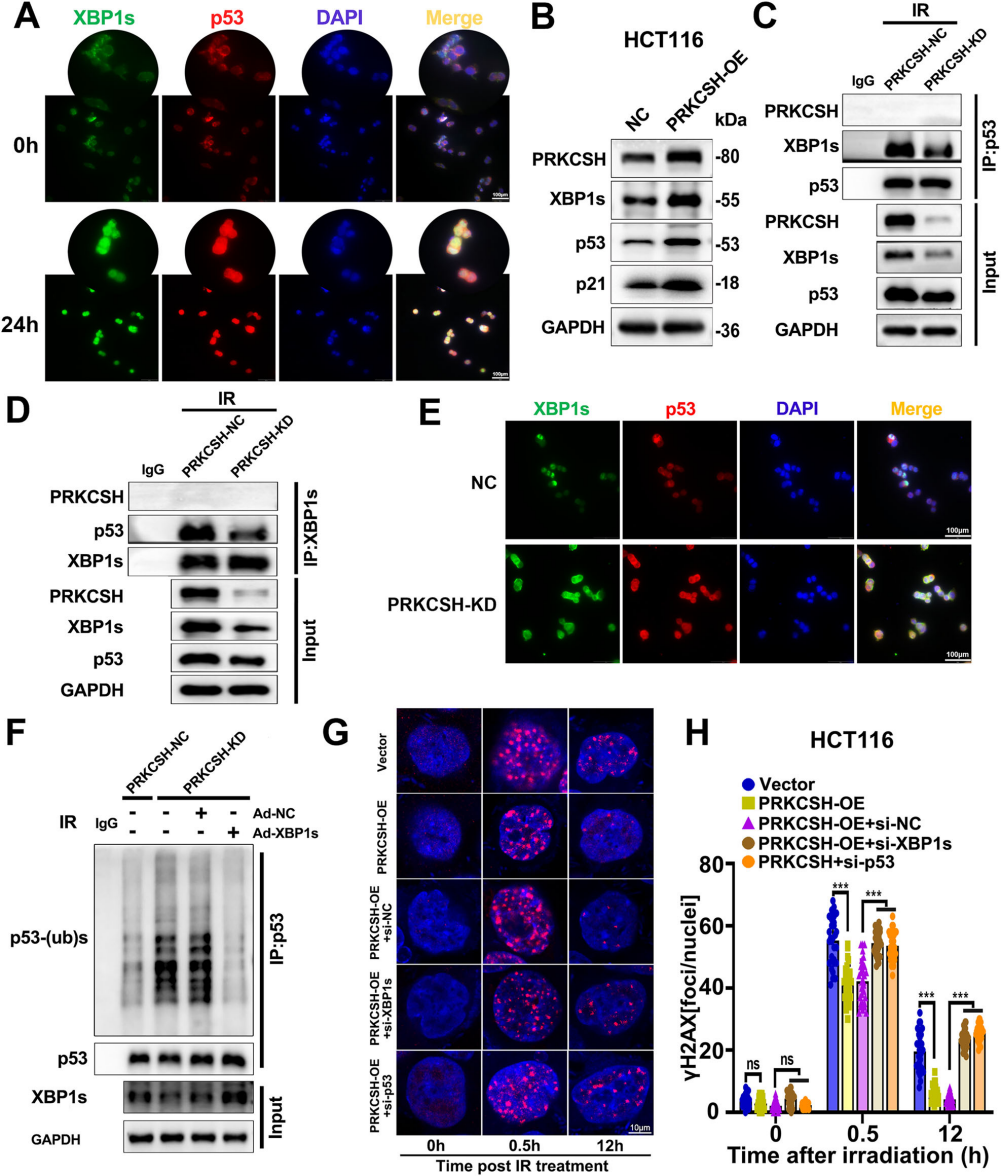
PRKCSH inhibits ubiquitinated degradation of p53 via XBP1s to promote DNA damage repair. **A** Immunofluorescence was employed to observe the expression and localization of XBP1s and p53 in HCT116 cells immediately and 24 h after 8 Gy irradiation. **B** PRKCSH, XBP1s, and p53 protein levels in PRKCSH-overexpressing HCT116 cells were measured by Western blot. **C** Immunoprecipitation of irradiated HCT116-NC and HCT116-PRKCSH-KD cell lysates revealed the co-precipitation of p53 with PRKCSH and XBP1s. **D** Similarly, XBP1s co-precipitated with PRKCSH and p53 post-irradiation. **E** Immunofluorescence staining showed the expression and localization of XBP1s (green) and p53 (red) in NC and PRKCSH-KD cells 24 h post-irradiation. **F** The impact of PRKCSH on p53 ubiquitination was assessed by Western blot. **G** DNA damage, indicated by γ-H2AX foci per cell, was measured in HCT116 cells transfected with Vector, PRKCSH-OE, PRKCSH-OE+si-NC, PRKCSH-OE+si-XBP1s, and PRKCSH-OE+si-p53 following IR exposure. **H** Quantitative analysis of γ-H2AX foci per cell was conducted across groups, with 30 cells per group at each time point. Error bars represent SD. “ns” indicates no significant difference; **P < 0.01, ***P < 0.001.

XBP1s is a key downstream effector in the IRE1α-mediated endoplasmic reticulum stress response. Under stress conditions, XBP1s translocates to the nucleus to regulate the expression of target proteins. For instance, Zhu et al. found that XBP1s interacts with p53, inhibiting its ubiquitination and subsequent degradation [20]. To observe the potential relationship between XBP1s and p53, we performed immunofluorescence to examine their localization before and after irradiation. In non-irradiated HCT116 cells, XBP1s and p53 primarily co-localized in the cytoplasm. However, after ionizing radiation, their co-localization shifted to the nucleus (Fig. 6A).

p53 plays a central role in enhancing DNA damage repair, which is crucial for resistance to radiotherapy [21]. Our previous research demonstrated that PRKCSH overexpression activates the IRE1α/XBP1s pathway in the endoplasmic reticulum stress response. To further investigate, we explored the impact of PRKCSH on the interaction between XBP1s and p53, as well as its regulation of p53 ubiquitination and stability. The results showed that PRKCSH overexpression significantly increased p53 protein levels (Fig. 6B). Immunoprecipitation analysis revealed that after PRKCSH knockdown, the interaction between p53 and XBP1s decreased in irradiated HCT116 cells (Fig. 6C, D). Immunofluorescence experiments further confirmed that PRKCSH knockdown significantly reduced the expression and co-localization of XBP1s and p53 (Fig. 6E). Additionally, in PRKCSH-knockdown cells, p53 ubiquitination levels increased, while XBP1s overexpression inhibited this effect (Fig. 6F). These findings suggest that under ionizing radiation, PRKCSH regulates p53 stability by modulating the interaction between XBP1s and p53. Given p53’s critical role in DNA damage repair, we also assessed DNA damage in irradiated HCT116 cells. PRKCSH overexpression significantly reduced radiation-induced DNA damage, but this effect was negated when p53 or XBP1s was further knocked down (Fig. 6G, H), indicating that PRKCSH promotes DNA damage repair by enhancing p53 stability. Furthermore, p53 knockdown reversed the effects of PRKCSH overexpression on cell cycle regulation and apoptosis following irradiation (Supplementary Fig. 4A–D).

Studies have shown that HT29 colorectal cancer cells exhibit greater radiation resistance compared to HCT116 cells [22, 23]. To investigate further, we examined the baseline expression levels of PRKCSH and p53 in these two cell lines. Our findings revealed that both PRKCSH and p53 were expressed at higher levels in HT29 cells compared to HCT116 cells (Supplementary Fig. 4E).

### Inhibition of PRKCSH enhances colorectal cancer radiosensitivity in vivo

**Fig. 7:**
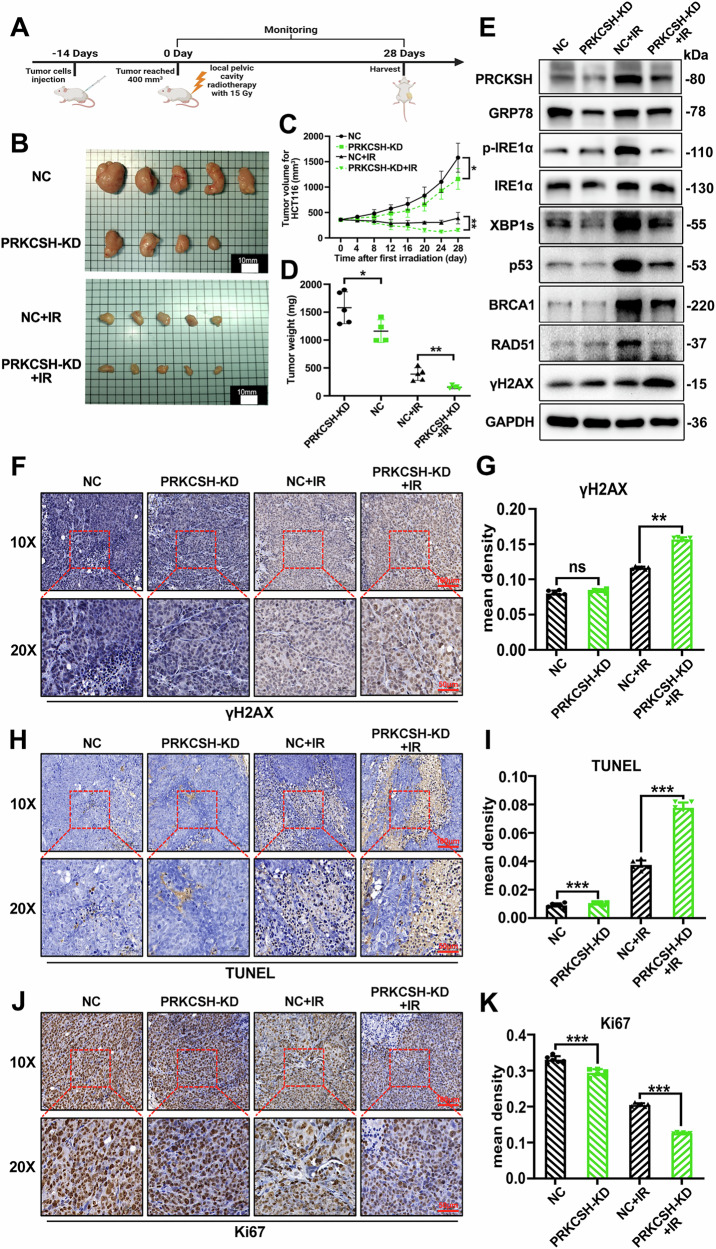
Inhibition of PRKCSH enhances the sensitivity of colorectal cancer xenograft tumor to radiation in vivo. **A** Overview of the animal experiment design. **B** Photographs of xenograft tumors taken 28 days after 15 Gy radiotherapy in the pelvic cavity. **C** Tumor volume growth in PRKCSH-NC and PRKCSH-KD groups, with and without ionizing radiation (IR). **D** Tumor weight measured at the time of sacrifice. **E** Western blot analysis of the IRE1α pathway and DNA damage repair signaling, showing data from a representative animal in each group. **F**–**J** Immunohistochemical images showing γH2AX, TUNEL, and Ki67 staining in tumor sections, based on representative tumors from each group. **G**–**K** Quantitative analysis of mean density for γH2AX, TUNEL, and Ki67 in tumors. Data are from three independent experiments. Error bars represent SD. ns indicates no significant difference; *P < 0.05, **P < 0.01, ***P < 0.001.

To investigate PRKCSH’s role in CRC radiotherapy, we established a CRC xenograft model by inoculating PRKCSH-knockdown (PRKCSH-KD) and control (NC) HCT116 cells into BALB/c nude mice. When tumors reached ~400 mm³, the mice received a single 15 Gy dose of abdominal radiation. Tumor growth was monitored every 4 days post-irradiation, and tumors were excised for analysis on day 28 (Fig. 7A). Following radiotherapy, tumors in the PRKCSH-NC group were larger than those in the PRKCSH-KD group. Without irradiation, the PRKCSH-NC group exhibited the fastest tumor growth, while PRKCSH-KD showed the most significant growth inhibition post-irradiation (Fig. 7B–D). These findings suggest that PRKCSH downregulation enhances CRC tumor radiosensitivity.

Western blot analysis of tumor samples revealed that PRKCSH knockdown inhibited the activation of the IRE1α/XBP1s pathway, reduced the expression of XBP1s and p53, and suppressed DNA damage repair mechanisms post-irradiation (Fig. 7E). Immunohistochemical analysis showed greater radiation-induced damage in PRKCSH-KD CRC cells compared to the control group (PRKCSH-NC) (Fig. 7F, G). TUNEL and Ki67 staining demonstrated increased apoptosis and reduced proliferation in PRKCSH-KD tumor cells (Fig. 7H–K).

To better mimic clinical treatment conditions, we established a tumor-bearing model in immunocompetent C57BL/6 mice and applied a fractionated irradiation regimen (8 Gy × 3), as described by Teng et al. [24]., to assess the role of PRKCSH. The results demonstrated that under this treatment regimen, the PRKCSH-knockdown group exhibited the most significant therapeutic effect (Supplementary Fig. 5A–C). These in vivo findings confirm that PRKCSH is a promising target for enhancing the radiosensitivity of colorectal cancer.

### High PRKCSH levels are associated with radioresistance in locally advanced rectal cancer patients treated with neoadjuvant radiotherapy

**Fig. 8:**
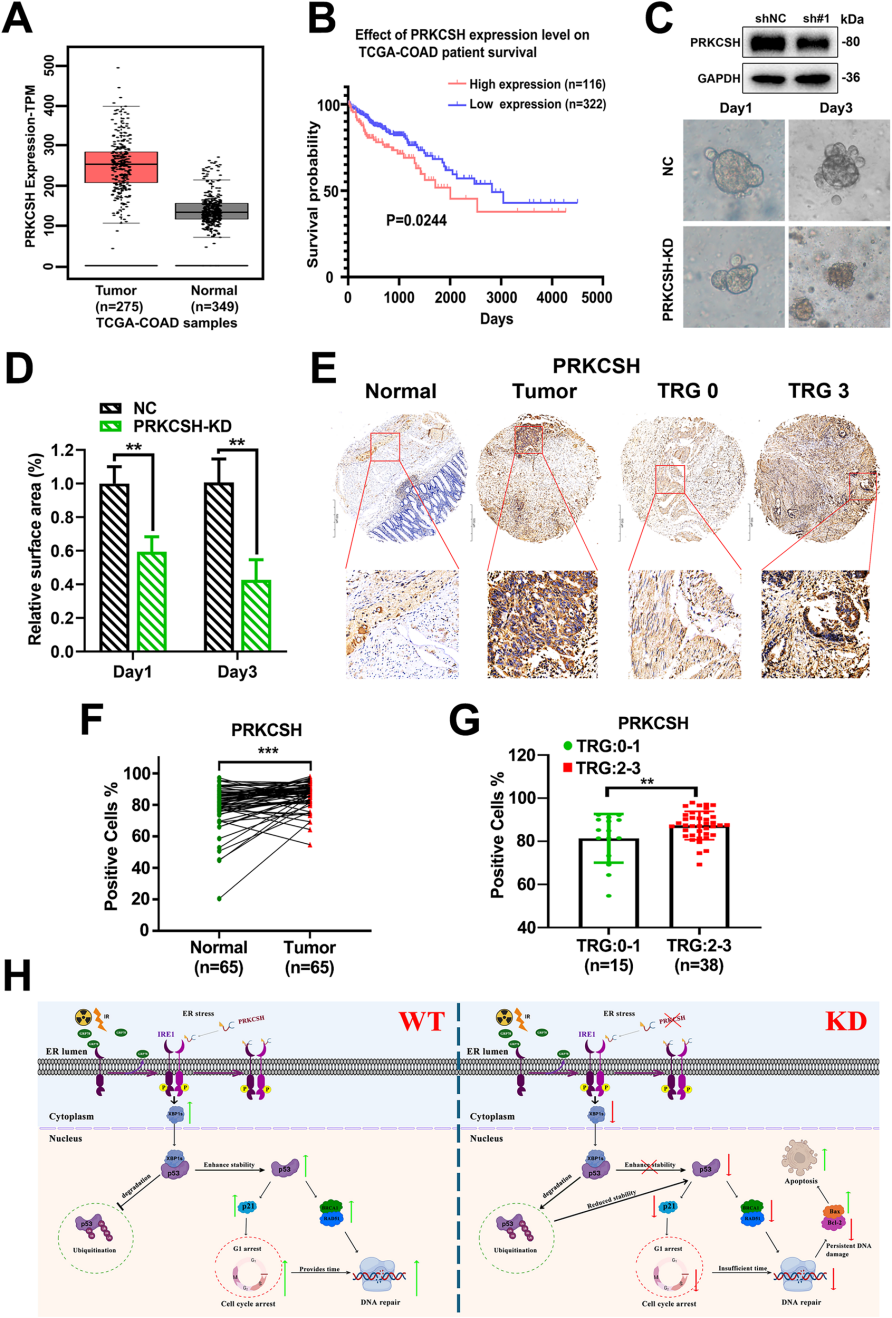
PRKCSH promotes radioresistance and poor prognosis in rectal cancer. **A** PRKCSH expression levels in colon adenocarcinoma (COAD) were analyzed using TCGA datasets, comparing normal tissue (*n* = 349) with colorectal adenocarcinoma tissue (*n* = 275). **B** A survival analysis of colorectal cancer patients was conducted based on PRKCSH expression, comparing high (*n* = 116) and low expression (*n* = 322) groups. **C** Representative patient-derived organoid (PDO) images showing tumor growth in NC and PRKCSH-KD groups at Day 1 and Day 3. PRKCSH knockdown validation was performed via Western blot (top). **D** Quantitative analysis of relative surface area of PDOs on Day 1 and Day 3. **E** Immunohistochemical (IHC) staining of PRKCSH in rectal cancer tissues, comparing radiosensitive (TRG = 0–1) and radioresistant (TRG = 2–3) tumors, as well as adjacent normal tissues. **F** Statistical analysis of PRKCSH-positive cell rates in tumors and adjacent normal tissues. **G** Comparison of PRKCSH-positive cell rates between radiotherapy-sensitive (TRG: 0–1) and resistant (TRG: 2–3) rectal cancer tissues. **H** PRKCSH was identified as a regulator of colorectal cancer radiosensitivity, promoting DNA damage repair via the IRE1α/XBP1s signaling pathway. Statistical significance is indicated by *P < 0.05, **P < 0.01, ***P < 0.001.

PRKCSH plays a crucial role in radiosensitization, with significant clinical implications. TCGA data analysis revealed that PRKCSH expression is elevated in colorectal adenocarcinoma (COAD) compared to normal tissues (Fig. 8A). Moreover, higher PRKCSH levels were inversely correlated with overall survival in COAD patients (Fig. 8B), suggesting that PRKCSH could serve as a prognostic marker for poor outcomes.

To assess PRKCSH’s preclinical relevance, we obtained clinical samples from the colorectal surgery department and developed a patient-derived organoid (PDO) model of rectal tumors. These organoids were transduced with lentiviruses expressing either NC or shPRKCSH and then exposed to 8 Gy ionizing radiation. A representative image of PDO growth is shown (Fig. 8C). Quantitative analysis of all PDOs revealed that PRKCSH knockdown significantly inhibited organoid growth, as indicated by a reduction in relative surface area on day 1 and day 3 post-irradiation compared to controls (Fig. 8D, *P* < 0.01). These findings underscore the pivotal role of PRKCSH in CRC tumor growth regulation. We also analyzed 53 patients who underwent neoadjuvant radiotherapy, classifying them into tumor regression grades (TRG0-3). TRG0-1 was categorized as radiosensitive, and TRG2-3 as radioresistant. Immunohistochemical staining revealed that PRKCSH expression was significantly upregulated in tumor tissues compared to adjacent normal tissues (Fig. 8E, F). Furthermore, PRKCSH expression was associated with rectal cancer radiosensitivity, showing a higher proportion of PRKCSH-positive cells in the radioresistant group (TRG2-3) compared to the radiosensitive group (TRG0-1) (Fig. 8E, G).

These findings suggest that PRKCSH contributes to radioresistance and may serve as a biomarker for monitoring tumor resistance or predicting recurrence after neoadjuvant chemoradiotherapy (CRT). Elevated PRKCSH levels in post-CRT tumor tissues reflect an adaptive tumor response to radiotherapy and correlate with poor tumor regression in advanced rectal cancer patients. Targeting PRKCSH could offer a promising strategy to overcome radioresistance and enhance the efficacy of radiotherapy in rectal cancer.

## Discussion

Colorectal cancer (CRC) is a major global health issue, causing ~700,000 deaths annually and ranking second in cancer-related mortality [25, 26]. Neoadjuvant radiotherapy effectively reduces local progression in advanced rectal cancer, but radioresistance remains a significant challenge in CRC treatment [27, 28]. This study identifies PRKCSH as a novel factor involved in the ER stress and DNA damage response in CRC. To our knowledge, this is the first study to demonstrate that PRKCSH enhances CRC radiosensitivity by modulating ER stress to improve DNA damage repair. Primarily located in the endoplasmic reticulum, PRKCSH plays a crucial role in protein synthesis and folding and is strongly linked to tumor development [14, 29–31]. However, evidence connecting PRKCSH to radiotherapy and DNA damage repair is currently limited.

Our data demonstrate that PRKCSH exhibits a time-dependent increase following ionizing radiation and is involved in IR-induced endoplasmic reticulum (ER) stress and DNA damage repair responses. Knocking down PRKCSH significantly attenuated the DNA damage response (DDR) in colorectal cancer (CRC) cells and xenograft tumor models, thereby enhancing CRC radiosensitivity. Mechanistically, PRKCSH was found to influence DNA damage repair by facilitating the interaction between XBP1s and p53, while preventing p53 degradation through ubiquitination. In clinical samples, PRKCSH was highly expressed in rectal cancer tissues, with lower expression in adjacent normal tissues. Notably, elevated PRKCSH levels were associated with increased tumor resistance to radiation therapy. All clinical samples in this study were collected after the completion of neoadjuvant chemoradiotherapy (CRT), reflecting post-treatment tumor characteristics rather than pre-treatment baseline levels. This distinction highlights PRKCSH’s potential role in treatment-induced resistance mechanisms and underscores its value as a biomarker for monitoring resistance or predicting recurrence following CRT.

Endoplasmic reticulum (ER) stress sensors and their associated signaling pathways play crucial roles in regulating tumor progression, metastasis, and responses to therapies such as chemotherapy, targeted treatments, and immunotherapies [32, 33]. Studies have shown that compounds like Bixin [34], Shikonin [35], Dehydrodiisoeugenol [36], and Cryptotanshinone [37] inhibit colorectal cancer (CRC) growth by inducing apoptosis or autophagy through ER stress. Additionally, ionizing radiation has been found to trigger an ER stress response [38–41], aligning with our findings that ER stress is activated following radiation exposure. Shin et al. (2019) further demonstrated that PRKCSH selectively activates the IRE1α branch of the ER stress response by enhancing IRE1α autophosphorylation and oligomerization, thereby promoting tumor cell adaptation to ER stress and driving tumorigenesis and progression [12]. Building on Shin et al.‘s research under non-radiotherapy conditions, our study is the first to establish PRKCSH’s pivotal role in linking ER stress to DNA repair under radiotherapy-induced conditions. Specifically, we revealed that PRKCSH stabilizes p53 via XBP1s in response to ionizing radiation, highlighting its role in integrating ER stress with the DNA damage response (DDR).

Recent studies have shown that under stress, XBP1s can translocate to the nucleus and interact with p53, inhibiting its ubiquitination and degradation [20]. Our findings confirm this interaction, demonstrating that PRKCSH knockdown reduces it, leading to increased p53 ubiquitination and decreased protein levels. In response to DNA damage, p53 acts as a crucial regulator, orchestrating DNA repair through both direct and indirect mechanisms. p53 directly influences DNA repair by modulating key homologous recombination (HR) molecules such as BRCA1 and RAD51 [42–44], while also indirectly facilitating repair by activating p21 to induce cell cycle arrest [21, 45], thereby providing sufficient time for damage resolution and maintaining genomic stability. In our study, PRKCSH knockdown was found to significantly reduce the expression of BRCA1 and RAD51, indicating that PRKCSH may promote DNA repair by sustaining p53 stability and its downstream repair pathways. Additionally, PRKCSH knockdown impaired radiation-induced G1-phase cell cycle arrest, further compromising the efficiency of DNA damage repair. These findings suggest that the DNA repair deficiencies observed in PRKCSH-knockdown cells result from a combination of disrupted cell cycle regulation and reduced expression of critical DNA repair molecules, highlighting the multifaceted roles of p53 in maintaining genomic stability in response to radiation.

Furthermore, while p53 is traditionally recognized as a key mediator of apoptosis in response to DNA damage, its role is highly context-dependent. Under conditions of mild-to-moderate DNA damage, p53 predominantly facilitates cell cycle arrest and DNA repair, whereas severe or irreparable damage leads to its activation of pro-apoptotic genes. In our study, PRKCSH knockdown led to decreased p53 levels but increased apoptosis, suggesting that PRKCSH stabilizes p53 in a way that favors DNA repair over apoptosis. When PRKCSH is absent, impaired repair results in excessive DNA damage accumulation, ultimately activating alternative apoptotic pathways, such as the mitochondrial apoptosis pathway, as evidenced by the observed Bax/Bcl-2 imbalance. This compensatory mechanism may explain why PRKCSH-knockdown cells exhibit enhanced radiosensitivity despite reduced p53 stability.

p53 plays a crucial role in cell cycle regulation and apoptosis, and its mutation is a key driver of CRC progression. In this study, we used three cell lines: HCT116 (p53 wild-type), RKO (p53 wild-type), and HT29 (p53 mutant). The results demonstrated that PRKCSH knockdown significantly enhanced radiosensitivity in p53 wild-type cells. Although PRKCSH knockdown also increased radiosensitivity in HT29 cells, we speculate that this effect may be related to the retained non-transcriptional regulatory functions of mutant p53. This hypothesis is supported by existing literature, which shows that R273H mutant p53 (HT29) can retain partial non-transcriptional functions under certain conditions, such as regulating downstream molecules involved in cell cycle arrest and apoptosis [46–48]. These findings further support the hypothesis that PRKCSH regulates radiosensitivity through a p53-dependent pathway and highlight the critical role of p53 status in CRC radioresistance.

Radioresistance is a critical challenge in cancer therapy, involving mechanisms such as DNA damage repair, cell cycle regulation, and ER stress. Unlike classical radioresistance factors such as ATM, ATR, and PARP that directly regulate DNA repair pathways, PRKCSH stabilizes p53 by activating the ER stress IRE1α/XBP1s pathway to enhance DNA repair. Targeting PRKCSH holds promise as a novel radiosensitization strategy. Specifically, developing small-molecule inhibitors or gene-targeting approaches, such as siRNA, against PRKCSH could effectively block its function, inhibit radiation-induced DNA repair, and enhance tumor cell radiosensitivity. Moreover, combining PRKCSH-targeting strategies with existing radiosensitizers or ER stress pathway inhibitors may produce synergistic effects, offering novel therapeutic approaches to overcome radioresistance.

This study establishes PRKCSH as a key regulator of ER stress and DNA repair through the IRE1α/XBP1s pathway, though some limitations persist. Further research is needed to clarify PRKCSH’s specific role in homologous recombination (HRR) and non-homologous end joining (NHEJ) using functional repair assays. Although patient-derived organoids (PDOs) were utilized to model PRKCSH-mediated radioresistance, further validation through histological assessments and DNA sequencing is required to confirm their accuracy in representing patient tumors. Additionally, the role of other IRE1α functions, such as RIDD, has yet to be investigated. This study does not provide a detailed analysis of the ATF6 pathway, including nuclear cleavage, which limits a full assessment of UPR branches in radiation-induced stress responses. Future research will bridge these gaps to deepen our understanding of PRKCSH’s role in UPR regulation. Despite these limitations, our findings highlight PRKCSH as a critical modulator of the IRE1α/XBP1s axis, providing valuable therapeutic insights for improving colorectal cancer radiosensitivity.

## Conclusions

Our study identifies PRKCSH as a radiation resistance factor in CRC cells, influencing DNA repair in both in vitro and xenograft models. PRKCSH induces endoplasmic reticulum stress post-radiation, stabilizes p53, and promotes DNA repair. Elevated PRKCSH expression is associated with poor tumor regression in locally advanced rectal cancer patients following neoadjuvant radiotherapy. These findings suggest that PRKCSH expression in post-treatment samples may serve as a biomarker for evaluating radiotherapy efficacy and guiding subsequent therapeutic strategies. Furthermore, targeting PRKCSH represents a promising approach for radiosensitization, with potential applications in improving the outcomes of precision medicine in clinical practice.

## Materials and methods

### Cell lines and cell culture

The HCT116, RKO, HT29, and MC38 colorectal cancer cell lines and the 293 T renal epithelial cell line were obtained from ATCC and cultured following their recommended protocols. HCT116 and HT29 cells were grown in McCoy’s 5 A medium, while RKO, MC38 and 293 T cells were cultured in DMEM, both supplemented with 10% FBS and 1% penicillin-streptomycin-glutamine. All cultures were incubated at 37 °C in a 5% CO_2_ atmosphere.

### Bioinformatics data analysis

The GEO dataset GSE10950 includes 24 paired colorectal cancer tumor and adjacent normal tissue samples, analyzed using the Illumina BeadChip Human Ref8-v2 platform. Data processing involved quantile normalization and log2 transformation for cross-sample comparability. UMAP was applied to visualize sample distribution and detect batch effects or outliers. Similarly, the GSE226034 dataset was analyzed to compare PRKCSH expression between radiation-resistant and radiation-sensitive groups after normalization. Additionally, mRNA sequencing data for COAD were retrieved from TCGA, including 275 COAD samples and 349 adjacent normal tissues. As publicly accessible data were utilized, ethical approval and informed consent were not required.

mRNA expression data from 701 colorectal cancer (CRC) samples (51 normal and 650 tumor tissues) were retrieved from TCGA (https://portal.gdc.cancer.gov/). Genes associated with the IRE1α/XBP1s signaling pathway and DNA damage repair were obtained from the MSigDB database (https://www.gsea-msigdb.org/) and published literature. Single-sample gene set enrichment analysis (ssGSEA) was performed to quantify pathway activity. Samples were grouped based on IRE1α/XBP1s expression levels, and DNA damage repair indicators were compared between high- and low-expression groups.

### Tissue sample sources and ethical approval

Tumor tissue samples were sourced from the specimen repository of Changhai Hospital, Shanghai, China. This study received ethical approval from the Changhai Hospital Ethics Committee, and all participants provided informed consent for the use of their tissue samples. A total of 12 rectal cancer patients, treated with neoadjuvant radiotherapy prior to surgery, were stratified into radiation-sensitive and radiation-resistant groups according to their tumor regression grade (TRG). Specifically, six patients were categorized as radiation-sensitive (TRG 0–1), while the remaining six were classified as radiation-resistant (TRG 2–3). TRG 0 indicates complete tumor regression, TRG 1 reflects near-complete regression with minimal residual tumor cells, TRG 2 represents partial regression with considerable residual tumor, and TRG 3 denotes minimal or no tumor regression.

### Stable cell line construction

Lentiviral packaging was performed by inoculating 293 T cells in the logarithmic growth phase into 10-cm culture dishes. PRKCSH-knockdown (sh-PRKCSH, Table S2) and PRKCSH-overexpression (PRKCSH-OE) plasmids were obtained from OBiO Technology (Shanghai, China). Lentiviral plasmids and packaging plasmids (pMD2.5 G and psPAX2) were co-transfected into 293 T cells using Lipofectamine 3000, following the manufacturer’s protocol. After 72 h of incubation, the supernatant was collected, centrifuged at 3000 rpm for 10 min, and filtered through a 0.45 μm membrane. Stable PRKCSH-OE cell lines were generated by infecting HCT116 cells with the purified PRKCSH-OE virus, while PRKCSH-KD cell lines were established by infecting HCT116, RKO and HT29 cells with the PRKCSH-KD virus. Viral infections were performed when cells reached 30–40% confluence, using polybrene (5 μg/mL) to enhance infection efficiency. Stable cell lines were selected with puromycin (2 μg/mL) for 96 h.

### Ionizing radiation (IR)

The experimental radiation was conducted at the University’s Irradiation Center. Cells were exposed to radiation doses of 2, 4, 6, and 8 Gy, following protocols established in previous studies [49]. Protein samples were collected at 0.5, 4, 8, 12, and 24 h post-irradiation. Gamma rays were delivered at a rate of 1 Gy/min from a ^60^Co source, with irradiation performed at room temperature.

### RNA isolation and real-time PCR

In CRC patient samples, RNA was extracted using the Solarbio kit (China) and reverse transcribed using the PrimeScript™ RT Master Mix (Takara, Tokyo, Japan). PCR amplification was conducted on the Roche Light Cycler 2.0 (Germany) with TB Green™ Premix Ex Taq™ (Takara, Kusatsu, Shiga, Japan). PRKCSH expression was assessed using the 2^-ΔΔCt^ method, with GAPDH as the internal control, and triplicate PCR amplifications were performed for each specimen.

### Western blotting

Following irradiation, proteins were extracted using M-PER Reagent with inhibitors, separated by SDS-PAGE, and transferred onto PVDF membranes. The membranes were blocked with non-fat milk, incubated with primary and secondary antibodies, and visualized using the ECL Western blotting method.

### Cell viability assay

Cell viability was assessed using the CCK-8 assay. Cells were plated in 96-well plates at a density of 3,000 cells per well and exposed to radiation doses of 0, 2, 4, or 8 Gy. CCK-8 solution (Dojindo, Japan) was added at 0, 24, 48, and 72 h post-irradiation, and absorbance was measured at 450 nm.

### Wound-healing assay

NC and PRKCSH-KD cells were cultured in 6-well plates until reaching 90–100% confluence. A wound was created using a pipette tip, and detached cells were removed with PBS. The cells were then exposed to 8 Gy radiation and incubated for 24 h. Wounds were imaged using a Nikon Ti inverted microscope, and wound widths were analyzed using ImageJ. The wound healing rate was calculated as [(initial wound width) - (wound width at specific intervals)]/(initial wound width) × 100%.

### Transwell invasion assay

Cell invasion was assessed using Transwell plates with 8 μm pores coated with Matrigel. Cells were starved, trypsinized, and seeded at a density of 4 × 10^4^ cells per well, with 10% FBS culture medium in the lower chamber. After 8 Gy irradiation and 24 h of incubation, non-invading cells were removed, and the remaining cells were fixed, stained, and examined under a microscope.

### Clonogenic survival assay

Cells were plated in 6-well plates at varying densities corresponding to radiation doses of 0, 2, 4, and 6 Gy, and incubated overnight. Following irradiation at 2, 4, or 6 Gy, the cells were cultured for 14 days until visible colonies formed. Colonies containing >50 cells were counted, and the survival rate was calculated as [(experimental group clones/seeded cells) / (control group clones/seeded cells)] × 100%.

### Apoptosis assay

Apoptotic activity was assessed using the Annexin V-APC/PI Apoptosis Detection Kit (Multi Sciences, China). Cells were collected at 0, 24, and 48 h post-irradiation, digested, and washed with PBS. After staining with Annexin V-APC and PI, the cells were analyzed by flow cytometry (Beckman, USA).

### Neutral comet assay

Slides were prepared by combining a 1% agarose and cell mixture, followed by overnight lysis in a neutral solution at 4°C. Electrophoresis was performed in TBE buffer at 1.0 V/cm for 20 min. The slides were then washed, treated with a DNA precipitation solution, and immersed in 70% ethanol, with each step lasting 20 min. After drying, the slides were stained with SYBR® Green Gold, rinsed, and analyzed under a fluorescence microscope. Tail moments of over 50 comets per slide were evaluated using CASP software.

### Immunofluorescence staining (IF)

Cells for γH2AX, XBP1s, and p53 immunostaining were cultured on chamber slides, then fixed, permeabilized, blocked, and incubated with primary and secondary antibodies. Fluorescence images were captured using a Zeiss LSM880 confocal microscope.

### Co-immunoprecipitation (Co-IP)

Cells were subjected to 8 Gy of radiation for 8 h, after which proteins were extracted. Protein co-immunoprecipitation was performed using antibodies for p53 or XBP1s, following the instructions provided in the Pierce™ Co-Immunoprecipitation Kit. The precipitated proteins were separated by SDS-PAGE and analyzed by immunoblotting with antibodies against PRKCSH, XBP1s, p53, and Ubiquitin.

### Model and local radiotherapy

This study complied with Chinese laboratory animal care guidelines (protocol number: SCXK [HU] 2017–0001) and was approved by the university’s ethics committee. Xenograft Tumor Model with Single-Dose Radiotherapy: Five-week-old male BALB/c nude mice were randomly assigned to four groups (*n* = 5 per group): NC, KD, NC + IR, and KD + IR. PRKCSH-NC and PRKCSH-KD HCT116 cells were subcutaneously injected into the inguinal region. Upon tumors reaching ~400 mm³, a single 15 Gy radiation dose was administered to the pelvic area. Tumor volumes were measured every 4 days, and mice were sacrificed after 28 days for tumor collection and analysis. Syngeneic Tumor Model with Fractionated Radiotherapy: Six-week-old male C57BL/6 mice were divided into four groups (*n* = 5 per group). Stable MC38-NC and MC38-PRKCSH-KD cells were subcutaneously injected. When tumors reached ~8 mm in diameter, fractionated radiotherapy was performed at 8 Gy per dose for three doses. Tumor volumes were measured every 3 days, and tumors were collected for analysis after 24 days. Tumor volume was calculated using the formula: *V* = 0.5 × a × b².

### Patient-derived organoids and treatment

Patient-derived organoid (PDO) models were established using colorectal cancer tissues from Shanghai Changhai Hospital. Tumor tissues were cut into ~5 mm³ pieces, digested at 37°C for 1 h, and centrifuged. The resulting pellet was treated with red blood cell lysis buffer, centrifuged again, resuspended, and combined with Matrigel. A 30 µL suspension was then dispensed into 48-well plates, followed by the addition of 500 µL organoid medium per well. The medium was changed every 2–3 days, and organoids were passaged every 5–7 days. NC and PRKCSH knockdown lentiviruses were introduced on day two post-passage. After 48 h, organoids were exposed to 8 Gy γ-rays, and images were captured using optical microscopy.

### IHC and TUNEL staining

Tumor tissue samples were fixed, embedded, sectioned (3 μm), dewaxed, and subjected to antigen retrieval. The sections were incubated with primary antibodies overnight at 4 °C, followed by secondary antibodies for 1 h at room temperature. They were then stained with DAB and hematoxylin, dehydrated, and air-dried. Immunostaining was performed using Ki67 and γH2AX antibodies, and apoptosis was assessed via TUNEL staining. Specimens were examined under a ZEISS microscope, and expression levels were quantified with Image-Pro software.

### Patients’ samples and tissue microarray

This study was approved by the Ethics Committee of Changhai Hospital, Naval Medical University (Shanghai, China). Clinical samples were collected from 65 patients with advanced rectal cancer who received neoadjuvant chemoradiotherapy (CRT) at the Department of Colorectal Surgery between May 2016 and October 2019. Eligible patients had a single primary tumor, completed standard neoadjuvant CRT, undergone surgical resection, and survived for at least 1 month after surgery.

Tumor and adjacent paracancerous tissues were collected during surgery following the completion of neoadjuvant CRT. The neoadjuvant CRT regimen consisted of a total radiotherapy dose of 50 Gy, delivered in 2 Gy increments using three-dimensional conformal radiotherapy (3DCRT) technology, combined with concurrent chemotherapy using capecitabine. After collection, the tissue samples were paraffin-embedded and processed into an immunohistochemistry (IHC) microarray for analysis (Supplementary Fig. 5D).

Tumor response to neoadjuvant CRT was evaluated using the tumor regression grade (TRG) system based on histopathological assessment of post-surgical specimens. TRG grades were defined as follows: TRG 0 indicates complete tumor regression, TRG 1 reflects near-complete regression with minimal residual tumor cells, TRG 2 represents partial regression with considerable residual tumor, and TRG 3 denotes minimal or no tumor regression. Patients were stratified into radiation-sensitive (TRG 0–1) and radiation-resistant (TRG 2–3) groups for further analysis.

### Statistical analysis

Statistical analyses were performed using unpaired two-tailed Student’s *t*-tests for comparisons between two groups, one-way ANOVA for comparisons among multiple groups, and paired *t*-tests for comparing PRKCSH expression between cancerous and non-cancerous tissues. Data are presented as means ± standard deviations (SD). Assumptions for these tests, including sample independence, homogeneity of variance, and normality, were presumed to be met. Statistical significance was defined as *P < 0.05, **P < 0.01, and ***P < 0.001. All analyses were conducted using GraphPad Prism 8 (GraphPad, USA).

## Supplementary information

Supplementary Information

Supplementary Figure 1

Supplementary Figure 2

Supplementary Figure 3

Supplementary Figure 4

Supplementary Figure 5

Full and uncropped western blots

## Data Availability

The datasets used and analyzed during the current study are available within the manuscript and its additional files.
