# Supplementary material for: PRKCSH enhances colorectal cancer radioresistance via IRE1α/XBP1s-mediated DNA repair
Source: Cell Death Dis. 2025 Apr 6;16(1):258. doi: 10.1038/s41419-025-07582-4 (PMC11973196; doi:10.1038/s41419-025-07582-4)
Supplement: Supplementary file 1 — Supplementary Information [file 41419_2025_7582_MOESM1_ESM.pdf]

Supplementary Figure 1 A-B. Western blot analysis confirmed PRKCSH expression levels in HT29 cells at various time points following 8 Gy irradiation. Quantitative gray value analysis is provided below the blots. **C.** Western blot analysis validated PRKCSH knockdown efficiency in HT29 cells using shRNA (sh#1) relative to the negative control (shNC). **D.** CCK-8 assay demonstrated cell proliferation in HT29 cells with PRKCSH knockdown (sh#1) and control (shNC), with or without 8 Gy irradiation, at the specified time points. **E.** Colony formation assay assessed the survival fraction of HT29 cells in PRKCSH knockdown (PRKCSH-KD) and control (NC) groups following irradiation doses ranging from 0 to 6 Gy. **F.** Quantitative analysis revealed a significant reduction in clonogenic survival in PRKCSH-KD cells compared to controls. **G.** Flow cytometry with Annexin V-APC/PI staining detected apoptosis in HT29 cells after 0 Gy and 8 Gy irradiation. **H.** Quantification of apoptotic cells at 0, 24, and 48 hours post-irradiation indicated a higher apoptosis rate in PRKCSH-KD cells compared to controls. Data are presented as mean ± SD from three independent experiments. Error bars represent SD. Statistical significance: *P < 0.05, **P < 0.01, ***P < 0.001.

Supplementary Figure 2 A. Expression levels of PRKCSH in radiosensitive and radioresistant tumor samples from GSE226034. **B.** Western blot analysis showing the expression levels of PRKCSH in HCT116 cells at 0, 24, 48, and 72 hours. **C.** Validation of PRKCSH overexpression in HCT116 cells. **D.** The proliferation of CRC cells in different groups after 8 Gy IR was determined by CCK-8 assay. **E.** Flow cytometry analysis of apoptosis in HCT116 cells with PRKCSH overexpression (OE) or vector control at 0, 24, and 48 hours post-irradiation. **F.** Representative images of Western Blot of the protein expression in apoptotic pathway, IRE1α/XBP1s pathway and DDR pathway in NC and PRKCSH-OE cells after 8 Gy IR at different time points. **G.** Relative ratio of Bcl2/Bax. **H.** Representative images from the comet assay of NC and OE cells at 8 h after 8 Gy IR. **I.** Tail moments in NC and OE cells were quantified using CASP 1.2.3b2 software. Statistical significance is indicated by *P < 0.05, **P < 0.01, ***P < 0.001.

Supplementary Figure 3 A-B. Clonogenic assay results showing the colony formation ability of HCT116 cells in the indicated groups (NC, PRKCSH-KD, PRKCSH-KD + Vector, and PRKCSH-KD + XBP1s) under 6 Gy irradiation. **C-D.** Invasion assay results showing the number of invasive cells in the indicated groups under 8 Gy irradiation. **E-F.** Wound-healing assay results showing the migration ability of cells in the indicated groups at 0 and 24 hours post-8 Gy irradiation. Data are presented as mean ± SD (n=3). **P < 0.01.

Supplementary Figure 4 A. Flow cytometry analysis of apoptosis was performed in the Vector, PRKCSH-OE, PRKCSH-OE + si-NC, and PRKCSH-OE + si-p53 groups at 0 and 24 hours post-irradiation. **B.** Quantification of apoptotic cells at the same time points showed a significant increase in apoptosis in the PRKCSH-OE group compared to the Vector group, an effect reversed by p53 knockdown. **C.** Flow cytometry was also used to assess cell cycle distribution under the same conditions as in panel A. PRKCSH overexpression induced G1 phase arrest, which was mitigated by p53 knockdown. **D.**

Quantitative analysis of cell cycle distribution at 0 and 24 hours post-irradiation revealed significant differences between the groups (***p < 0.001). **E.** Western blot analysis confirmed the expression levels of PRKCSH and p53 in HCT116 and HT29 cells. Statistical significance is indicated by *P < 0.05, **P < 0.01, ***P < 0.001.

Supplementary Figure 5 A. Schematic representation of the experimental timeline. C57BL/6 mice were subcutaneously injected with MC38 cells, and when tumors reached approximately 8 mm in diameter, they received 8 Gy radiation therapy (RT) on days 0, 1, and 2. **B.** Photographs of xenograft tumors taken 24 days post-localized fractionated radiation therapy. The experimental groups were NC, KD, NC+IR, and KD+IR. **C.** Tumor growth curves over time demonstrated a significant reduction in tumor volume in the KD+IR group compared to the NC+IR group (**p < 0.01). **D.** Tissue microarray immunohistochemistry was used to evaluate PRKCSH protein expression in tumor and adjacent normal tissues from clinical rectal cancer patients. T represents tumor, and N represents normal tissue. Statistical significance is indicated by *P < 0.05, **P < 0.01, ***P < 0.001.

Table S1. The H-score of each sample in the tissue microarray

Number	Names of Images	TRG	Percentage of positive cells, %
1	CRC 202010 PRKCSH-A01	2	87.51%
2	CRC 202010 PRKCSH-A02		91.95%
3	CRC 202010 PRKCSH-A03	0	92.17%
4	CRC 202010 PRKCSH-A04		82.24%
5	CRC 202010 PRKCSH-A05	3	97.95%
6	CRC 202010 PRKCSH-A06		82.01%
7	CRC 202010 PRKCSH-A07	not found	94.07%
8	CRC 202010 PRKCSH-A08		94.89%
9	CRC 202010 PRKCSH-A09	3	91.32%
10	CRC 202010 PRKCSH-A10		87.54%
11	CRC 202010 PRKCSH-A11		95.31%
12	CRC 202010 PRKCSH-A13	2	79.66%
13	CRC 202010 PRKCSH-A14		58.55%
14	CRC 202010 PRKCSH-B01	3	96.71%
15	CRC 202010 PRKCSH-B02		84.97%
16	CRC 202010 PRKCSH-B03	0	81.40%
17	CRC 202010 PRKCSH-B04		97.16%
18	CRC 202010 PRKCSH-B06		86.25%
19	CRC 202010 PRKCSH-B07	2	82.79%
20	CRC 202010 PRKCSH-B08		78.10%
21	CRC 202010 PRKCSH-B09	3	96.86%
22	CRC 202010 PRKCSH-B10		81.85%
23	CRC 202010 PRKCSH-B11	2	90.66%

24	CRC 202010 PRKCSH-B12		82.56%
25	CRC 202010 PRKCSH-B13	3	81.39%
26	CRC 202010 PRKCSH-B14		80.47%
27	CRC 202010 PRKCSH-B15	3	87.45%
28	CRC 202010 PRKCSH-B16		52.83%
29	CRC 202010 PRKCSH-C01	3	97.13%
30	CRC 202010 PRKCSH-C02		79.92%
31	CRC 202010 PRKCSH-C03	1	92.24%
32	CRC 202010 PRKCSH-C04		92.31%
33	CRC 202010 PRKCSH-C05	3	69.26%
34	CRC 202010 PRKCSH-C06		79.37%
35	CRC 202010 PRKCSH-C07	3	80.63%
36	CRC 202010 PRKCSH-C08		91.67%
37	CRC 202010 PRKCSH-C09	3	93.39%
38	CRC 202010 PRKCSH-C10		86.22%
39	CRC 202010 PRKCSH-C12		87.92%
40	CRC 202010 PRKCSH-C13	1	90.00%
41	CRC 202010 PRKCSH-C14		75.56%
42	CRC 202010 PRKCSH-C15	0	69.17%
43	CRC 202010 PRKCSH-C16		20.51%
44	CRC 202010 PRKCSH-D01	1	91.16%
45	CRC 202010 PRKCSH-D02		95.54%
46	CRC 202010 PRKCSH-D03	3	90.13%
47	CRC 202010 PRKCSH-D04		89.23%
48	CRC 202010 PRKCSH-D05	3	88.27%
49	CRC 202010 PRKCSH-D06		81.25%
50	CRC 202010 PRKCSH-D07	2	86.90%
51	CRC 202010 PRKCSH-D08		85.77%
52	CRC 202010 PRKCSH-D09	not found	89.51%
53	CRC 202010 PRKCSH-D10		84.32%
54	CRC 202010 PRKCSH-D11	1	84.89%
55	CRC 202010 PRKCSH-D12		95.24%
56	CRC 202010 PRKCSH-D15	1	81.45%
57	CRC 202010 PRKCSH-D16		45.13%
58	CRC 202010 PRKCSH-E01		84.67%
59	CRC 202010 PRKCSH-E03	2	95.42%
60	CRC 202010 PRKCSH-E04		80.46%
61	CRC 202010 PRKCSH-E05	2	75.49%
62	CRC 202010 PRKCSH-E06		50.70%
63	CRC 202010 PRKCSH-E07	2	87.06%
64	CRC 202010 PRKCSH-E08		88.42%
65	CRC 202010 PRKCSH-E09	1	85.26%
66	CRC 202010 PRKCSH-E10		74.10%
67	CRC 202010 PRKCSH-E11	2	90.88%

68	CRC 202010 PRKCSH-E12		86.54%
69	CRC 202010 PRKCSH-E14		65.35%
70	CRC 202010 PRKCSH-E16		70.25%
71	CRC 202010 PRKCSH-F01	2	86.94%
72	CRC 202010 PRKCSH-F02		90.37%
73	CRC 202010 PRKCSH-F03	1	54.73%
74	CRC 202010 PRKCSH-F04		69.72%
75	CRC 202010 PRKCSH-F05	1	81.50%
76	CRC 202010 PRKCSH-F06		75.58%
77	CRC 202010 PRKCSH-F07	3	84.77%
78	CRC 202010 PRKCSH-F08		87.90%
79	CRC 202010 PRKCSH-F11	2	83.45%
80	CRC 202010 PRKCSH-F12		69.52%
81	CRC 202010 PRKCSH-F13	2	90.28%
82	CRC 202010 PRKCSH-F14		85.10%
83	CRC 202010 PRKCSH-G01	2	93.49%
84	CRC 202010 PRKCSH-G02		73.51%
85	CRC 202010 PRKCSH-G06		86.44%
86	CRC 202010 PRKCSH-G07	1	89.87%
87	CRC 202010 PRKCSH-G08		78.99%
88	CRC 202010 PRKCSH-G09	2	92.22%
89	CRC 202010 PRKCSH-G10		80.87%
90	CRC 202010 PRKCSH-G11	2	87.09%
91	CRC 202010 PRKCSH-G12		77.61%
92	CRC 202010 PRKCSH-G13	2	86.02%
93	CRC 202010 PRKCSH-G14		44.75%
94	CRC 202010 PRKCSH-G15	3	85.18%
95	CRC 202010 PRKCSH-G16		66.01%
96	CRC 202010 PRKCSH-H01	2	96.40%
97	CRC 202010 PRKCSH-H02		85.21%
98	CRC 202010 PRKCSH-H03	not found	82.32%
99	CRC 202010 PRKCSH-H04		79.97%
100	CRC 202010 PRKCSH-H05	3	81.56%
101	CRC 202010 PRKCSH-H06		78.50%
102	CRC 202010 PRKCSH-H07	2	74.51%
103	CRC 202010 PRKCSH-H08		69.08%
104	CRC 202010 PRKCSH-H09	2	88.43%
105	CRC 202010 PRKCSH-H10		86.22%
106	CRC 202010 PRKCSH-H11	0	64.34%
107	CRC 202010 PRKCSH-H12		77.93%
108	CRC 202010 PRKCSH-H13	1	73.33%
109	CRC 202010 PRKCSH-H14		76.66%
110	CRC 202010 PRKCSH-H15	0	89.32%
111	CRC 202010 PRKCSH-H16		78.18%

112	CRC 202010 PRKCSH-I01	2	88.66%
113	CRC 202010 PRKCSH-I02		87.39%
114	CRC 202010 PRKCSH-I03	2	87.59%
115	CRC 202010 PRKCSH-I04		82.08%
116	CRC 202010 PRKCSH-I05	2	82.44%
117	CRC 202010 PRKCSH-I06		77.05%
118	CRC 202010 PRKCSH-I07	2	82.93%
119	CRC 202010 PRKCSH-I08		75.97%
120	CRC 202010 PRKCSH-I09	not found	95.93%
121	CRC 202010 PRKCSH-I10		78.58%
122	CRC 202010 PRKCSH-I11	not found	74.28%
123	CRC 202010 PRKCSH-I12		76.30%
124	CRC 202010 PRKCSH-I13	not found	87.20%
125	CRC 202010 PRKCSH-I14		66.01%
126	CRC 202010 PRKCSH-I15	not found	85.50%
127	CRC 202010 PRKCSH-I16		83.59%
128	CRC 202010 PRKCSH-J01	not found	94.46%
129	CRC 202010 PRKCSH-J02		91.64%
130	CRC 202010 PRKCSH-J03	not found	80.66%
131	CRC 202010 PRKCSH-J04		80.85%
132	CRC 202010 PRKCSH-J05	not found	88.66%
133	CRC 202010 PRKCSH-J06		83.72%
134	CRC 202010 PRKCSH-J07	not found	84.96%
135	CRC 202010 PRKCSH-J08		86.81%
136	CRC 202010 PRKCSH-J09	not found	75.53%
137	CRC 202010 PRKCSH-J10		89.00%
138	CRC 202010 PRKCSH-J11	not found	0.8395
139	CRC 202010 PRKCSH-J12	not found	0.7652
140	CRC 202010 PRKCSH-J13	not found	0.7976

Note: The "percentage of positive cells" indicates the proportion of cells showing positive staining relative to the total analyzed cells. It is calculated using the formula: Percentage of positive cells = (Number of positive cells / Total number of cells) × 100%. Positive cells are identified based on target protein or marker expression detected by immunohistochemistry (IHC).

Table S2. Sequence information used in this study

ID (human)	Sequence
PRKCSH sh1	GGAAGAAGAGGCTGAAGAA
PRKCSH sh2	GCGTTTCAGGCAGCTGTTT
shNC	CCTAAGGTTAAGTCGCCCTCG
**ID (human)**	**Sequence**
PRKCSH OE	ATGCTGTTGCCGCTGCTGCTGCTGCTACCCATGT<br>GCTGGGCCGTGGAGGTCAAGAGGCCCCGGGGCG<br>TCTCCCTCACCAATCATCACTTCTACGATGAGTCC

	AAGCCTTTCACCTGCCTGGACGGTTCGGCCACC<br>ATCCCATTTGATCAGGTCAACGATGACTATTGCG<br>ACTGCAAAGATGGCTCTGACGAGCCAGGCACGG<br>CTGCCTGTCCTAATGGCAGCTTCCACTGCACCAA<br>CACTGGCTATAAGCCCCTGTATATCCCCTCCAACC<br>GGGTCAACGATGGTGTTTGTGACTGCTGCGATGG<br>AACAGACGAGTACAACAGCGGCGTCATCTGTGA<br>GAACACCTGCAAAGAGAAGGGCCGTAAGGAGA<br>GAGAGTCCCTGCAGCAGATGGCCGAGGTCACCC<br>GCGAAGGGTTCCGTCTGAAGAAGATCCTTATTGA<br>GGACTGGAAGAAGGCACGGGAGGAGAAGCAGA<br>AAAAGCTCATTGAGCTACAGGCTGGGAAGAAGT<br>CTCTGGAAGACCAGGTGGAGATGCTGCGGACAG<br>TGAAGGAGGAAGCTGAGAAGCCAGAGAGAGAG<br>GCCAAAGAGCAGCACCAGAAGCTGTGGGAAGA<br>GCAGCTGGCTGCTGCCAAGGCCCAACAGGAGCA<br>GGAGCTGGCGGCTGATGCCTTCAAGGAGCTGGA<br>TGATGACATGGACGGGACGGTCTCGGTGACTGA<br>GCTGCAGACTCACCCGGAGCTGGACACAGATGG<br>GGATGGGGCGTTGTCAGAAGCGGAAGCTCAGGC<br>CCTCCTCAGTGGGGACACACAGACAGACGCCAC<br>CTCTTTCTACGACCGCGTCTGGGCCGCCATCAGG<br>GACAAGTACCGGTCCGAGGCACTGCCCACCGAC<br>CTTCCAGCACCTTCTGCCCCTGACTTGACGGAGC<br>CCAAGGAGGAGCAGCCGCCAGTGCCCTCGTCGC<br>CCACAGAGGAGGAGGAGGAGGAGGAGGAGGAG<br>GAGGAAGAAGAGGCTGAAGAAGAGGAGGAGGA<br>GGAGGATTCCGAGGTGCAGGGGGAGCAGCCCAA<br>GCCGGCCAGCCCTGCTGAGGAAGACAAAATGCC<br>GCCCTACGACGAGCAGACGCAGGCCTTCATCGAT<br>GCTGCCCAGGAGGCCCGCAACAAGTTCGAGGAG<br>GCCGAGCGGTCGCTGAAGGACATGGAGGAGTCC<br>ATCAGGAACCTGGAGCAAGAGATTTCTTTTGACT<br>TTGGCCCCAACGGGGAGTTTGCTTACCTGTACAG<br>CCAGTGCTACGAGCTCACCACCAACGAATACGTC<br>TACCGCCTCTGCCCCTTCAAGCTTGTCTCGCAGA<br>AACCCAAACTCGGGGGCTCTCCCACCAGCCTTG<br>GCACCTGGGGCTCATGGATTGGCCCCGACCACG<br>ACAAGTTCAGTGCCATGAAGTATGAGCAAGGCA<br>CGGGCTGCTGGCAGGGCCCCAACCGCTCCACCA<br>CCGTGCGCCTCCTGTGCGGGAAAGAGACCATGG<br>TGACCAGCACCACAGAGCCCAGTCGCTGCGAGT<br>ACCTCATGGAGCTGATGACGCCAGCCGCCTGCCC<br>GGAGCCACCGCCTGAAGCACCCACCGAAGACGA

	CCATGACGAGCTCTAG
**ID (mouse)**	**Sequence**
PRKCSH sh	CGACGACTACTGCGACTGTAA
shNC	TTCTCCGAACGTGTCACGTAA
**ID (human)**	**Sequence**
Ad-XBP1s	ATGGTGGTGGTGGCAGCCGCGCCGAACCCGGCC GACGGGACCCCTAAAGTTCTGCTTCTGTCGGGGC AGCCCGCCTCCGCCGCCGGAGCCCCGGCCGGCC AGGCCCTGCCGCTCATGGTGCCAGCCCAGAGAG GGGCCAGCCCGGAGGCAGCGAGCGGGGGGCTG CCCCAGGCGCGCAAGCGACAGCGCCTCACGCAC CTGAGCCCCGAGGAGAAGGCGCTGAGGAGGAA ACTGAAAAACAGAGTAGCAGCTCAGACTGCCAG AGATCGAAAGAAGGCTCGAATGAGTGAGCTGGA ACAGCAAGTGGTAGATTTAGAAGAAGAGAACCA AAAACTTTTGCTAGAAAATCAGCTTTTACGAGAG AAAACTCATGGCCTTGTAGTTGAGAACCAGGAG TTAAGACAGCGCTTGGGGATGGATGCCCTGGTTG CTGAAGAGGAGGCGGAAGCCAAGGGGAATGAA GTGAGGCCAGTGGCCGGGTCTGCTGAGTCCGCA GCAGGTGCAGGCCCAGTTGTCACCCCTCCAGAA CATCTCCCCATGGATTCTGGCGGTATTGACTCTTC AGATTCAGAGTCTGATATCCTGTTGGGCATTCTG GACAACTTGGACCCAGTCATGTTCTTCAAATGCC CTTCCCCAGAGCCTGCCAGCCTGGAGGAGCTCC CAGAGGTCTACCCAGAAGGACCCAGTTCCTTAC CAGCCTCCCTTTCTCTGTCAGTGGGGACGTCATC AGCCAAGCTGGAAGCCATTAATGAACTAATTCGT TTTGACCACATATATACCAAGCCCCTAGTCTTAGA GATACCCTCTGAGACAGAGAGCCAAGCTAATGT GGTAGTGAAAATCGAGGAAGCACCTCTCAGCCC CTCAGAGAATGATCACCCTGAATTCATTGTCTCA GTGAAGGAAGAACCTGTAGAAGATGACCTCGTT CCGGAGCTGGGTATCTCAAATCTGCTTTCATCCA GCCACTGCCCAAAGCCATCTTCCTGCCTACTGGA TGCTTACAGTGACTGTGGATACGGGGGTTCCCTT TCCCCATTCAGTGACATGTCCTCTCTGCTTGGTGT AAACCATTCTTGGGAGGACACTTTTGCCAATGAA CTCTTTCCCCAGCTGATTAGTGTCTAA
**Primer name**	**Sequence**
PRKCSH forward	5′-TCAGGTCAACGATGACTATTGC-3′
PRKCSH reverse	5′-GAAGGCTGGGGCTCATTT-3′
DGAT2 forward	5′-ATTGCTGGCTCATCGCTGT-3′
DGAT2 reverse	5′-GGGAAAGTAGTCTCGAAAGTAGC-3′

GAPDH forward	5′-CAGGAGGCATTGCTGATGAT-3′
GAPDH reverse	5′-GAA GGC TGG GGC TCA TTT-3′
**siRNA**	**Sequence**
IRE1α sense	5'-GAAUCCUCUACAUGGGUAAAAAGCATT-3'
IRE1α antisense	5'-UCUCCCAUAAUCCACUUUCTT-3'
p53 sense	5'-GUACCACCAUCCACUACAATT-3'
p53 antisense	5'-UUGUAGUGGAUGGUGGUACTT-3'
NC sense	5'-UUCUCCGAACGUGUCACGUTT-3'
NC antisense	5'-ACGUGACACGUUCGGAGAATT-3'

Table S3 Primary antibodies used in this study

Antibody	Application	Company	Catalog numbers
PRKCSH	1:2000 for WB	Proteintech	12148-1-AP
GRP78	1:2000 for WB	Proteintech	11587-1-AP
IRE1α	1:1000 for WB	Proteintech	27528-1-AP
p-IRE1α（S724）	1:1000 for WB	Abcam	ab124945
XBP1s	1:1000 for WB	Proteintech	24868-1-AP
XBP1s	1:500 for IF	Proteintech	24868-1-AP
XBP1s	1:1000 for IP	Abcam	ab220783
p53	1:200 for WB	Santa Cruz	sc-126
p53	1:500 for IF	Santa Cruz	sc-126
p53	1-2 μg per 100-500 μg of total protein for IP	Santa Cruz	sc-126
ub	1:1000 for WB	CST	58395
IgG	1:1000 for WB	CST	7074P2
IgG	1:2000 for WB	CST	91196
C-Caspase3	1:1000 for WB	CST	#9664
Bax	1:1000 for WB	CST	5023T
Bcl2	1:1000 for WB	CST	3498S
p-DNAPKcs(Ser2056)	1:1000 for WB	CST	68716S
BRCA1	1:1000 for WB	CST	9010S
P21	1:1000 for WB	Abcam	Ab109199
p-CDC2	1:1000 for WB	CST	4539s
CDC2	1:1000 for WB	CST	77055s
CyclinB1	1:1000 for WB	Proteintech	55004-1- AP
p-RPA2(phospho S33)	1:1000 for WB	Abcam	ab211877
RPA2	1:1000 for WB	Abcam	ab76420
RAD51	1:10000 for WB	Abcam	ab133534
γH2AX(Ser139)	1:1000 for WB	CST	80312s
γH2AX(Ser139)	1:500 for IF	CST	80312s
γH2AX(Ser139)	1:300 for IHC	Abcam	ab26350
GAPDH	1:5000 for WB	Proteintech	60004-1-Ig
Ki67	0.1 - 5 μg/ml for IHC	Abcam	ab15580

PERK	1:1000 for WB	Proteintech	20582-1-AP
p-PERK	1:1000 for WB	Proteintech	29546-1-AP
ATF6	1:2000 for WB	Proteintech	24169-1-AP
HRP-conjugated goat anti-mouse secondary antibody	1:5000 for WB	Servicebio	GB23301
HRP-conjugated goat anti-rabbit secondary antibody	1:5000 for WB	Servicebio	GB23303
