# Supplementary figures and images for: PRKCSH enhances colorectal cancer radioresistance via IRE1α/XBP1s-mediated DNA repair
Source: Cell Death Dis. 2025 Apr 6;16(1):258. doi: 10.1038/s41419-025-07582-4 (PMC11973196; doi:10.1038/s41419-025-07582-4)

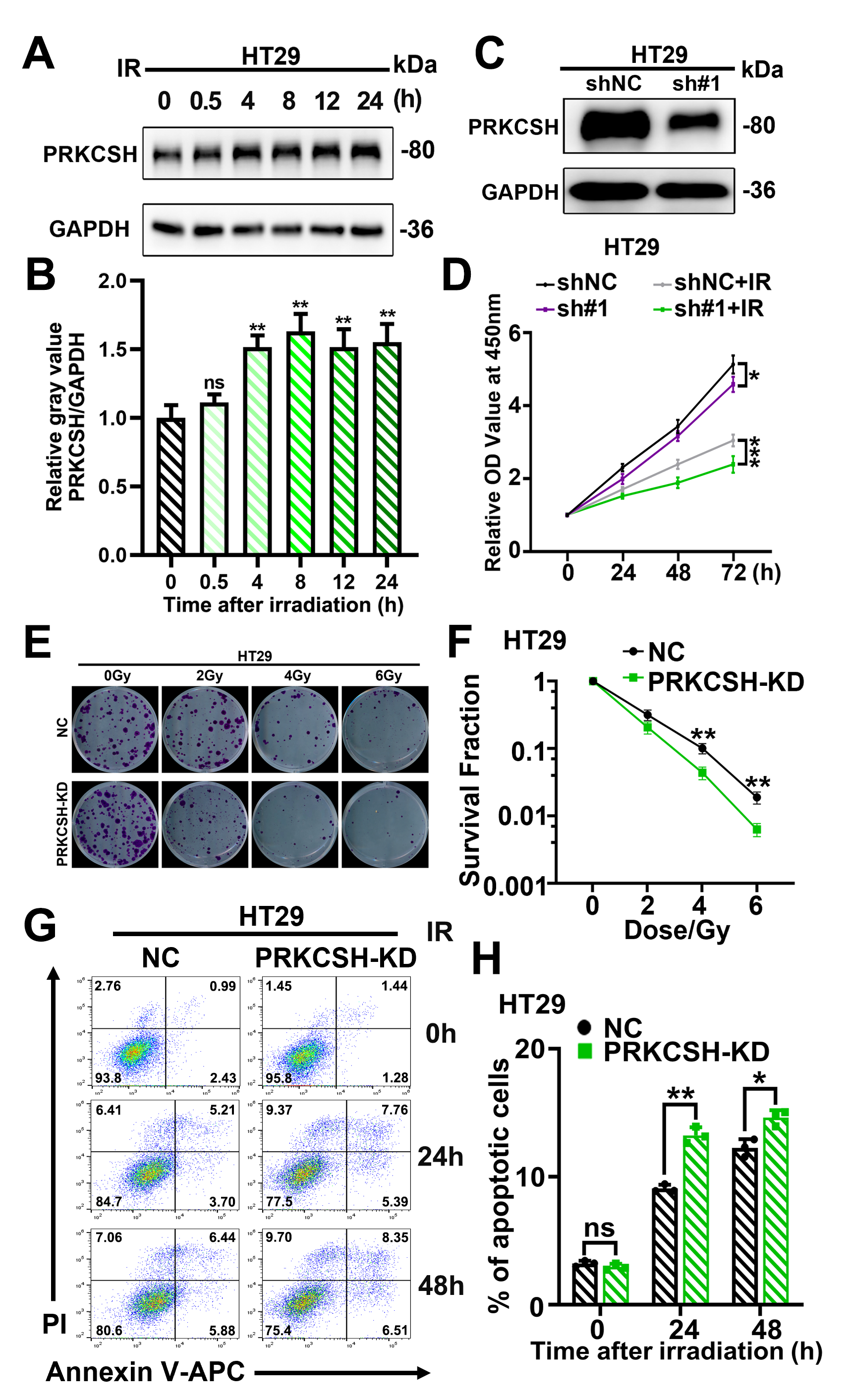

Supplement: Supplementary file 2 — Supplementary Figure 1 [file 41419_2025_7582_MOESM2_ESM.tif]

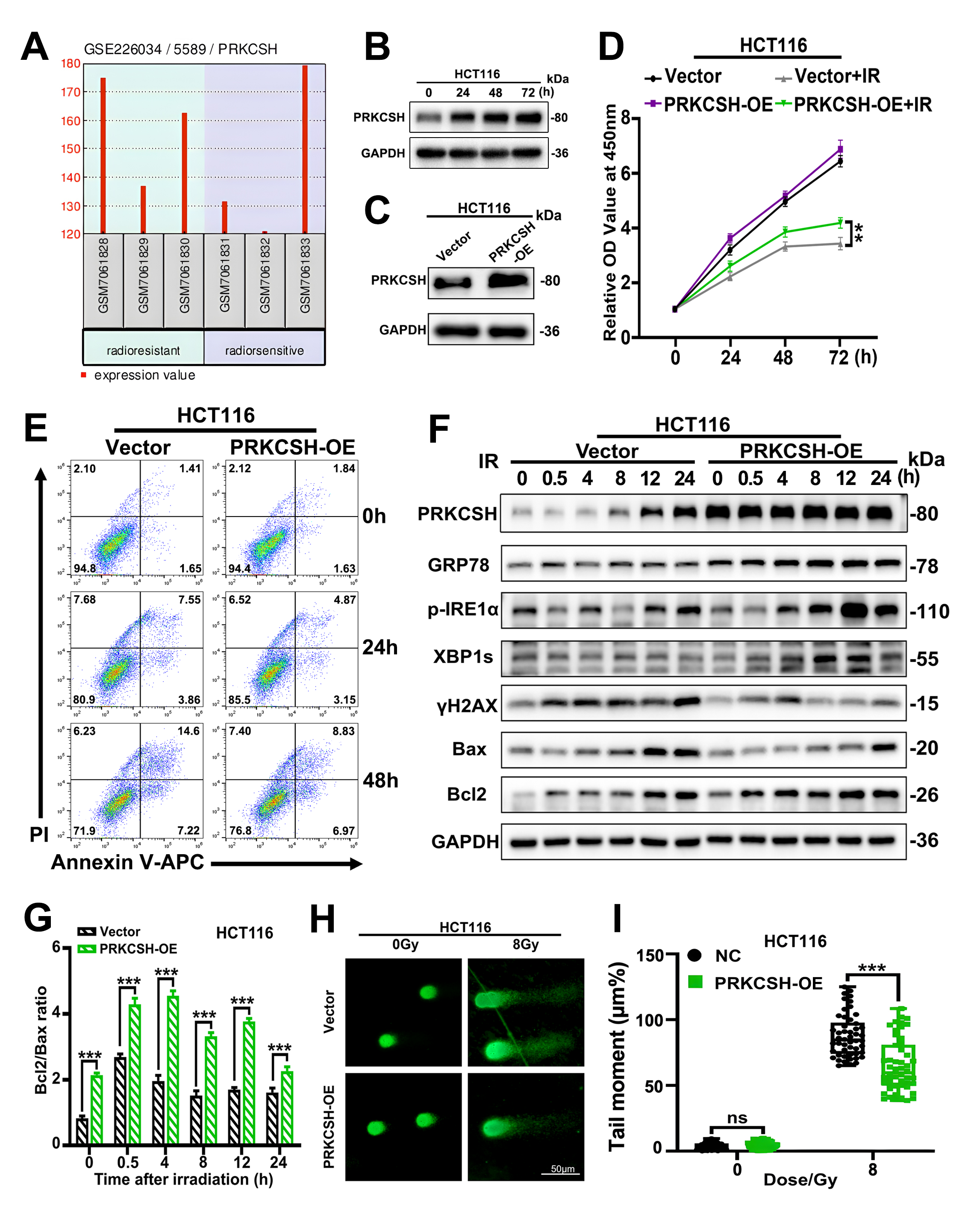

Supplement: Supplementary file 3 — Supplementary Figure 2 [file 41419_2025_7582_MOESM3_ESM.tif]

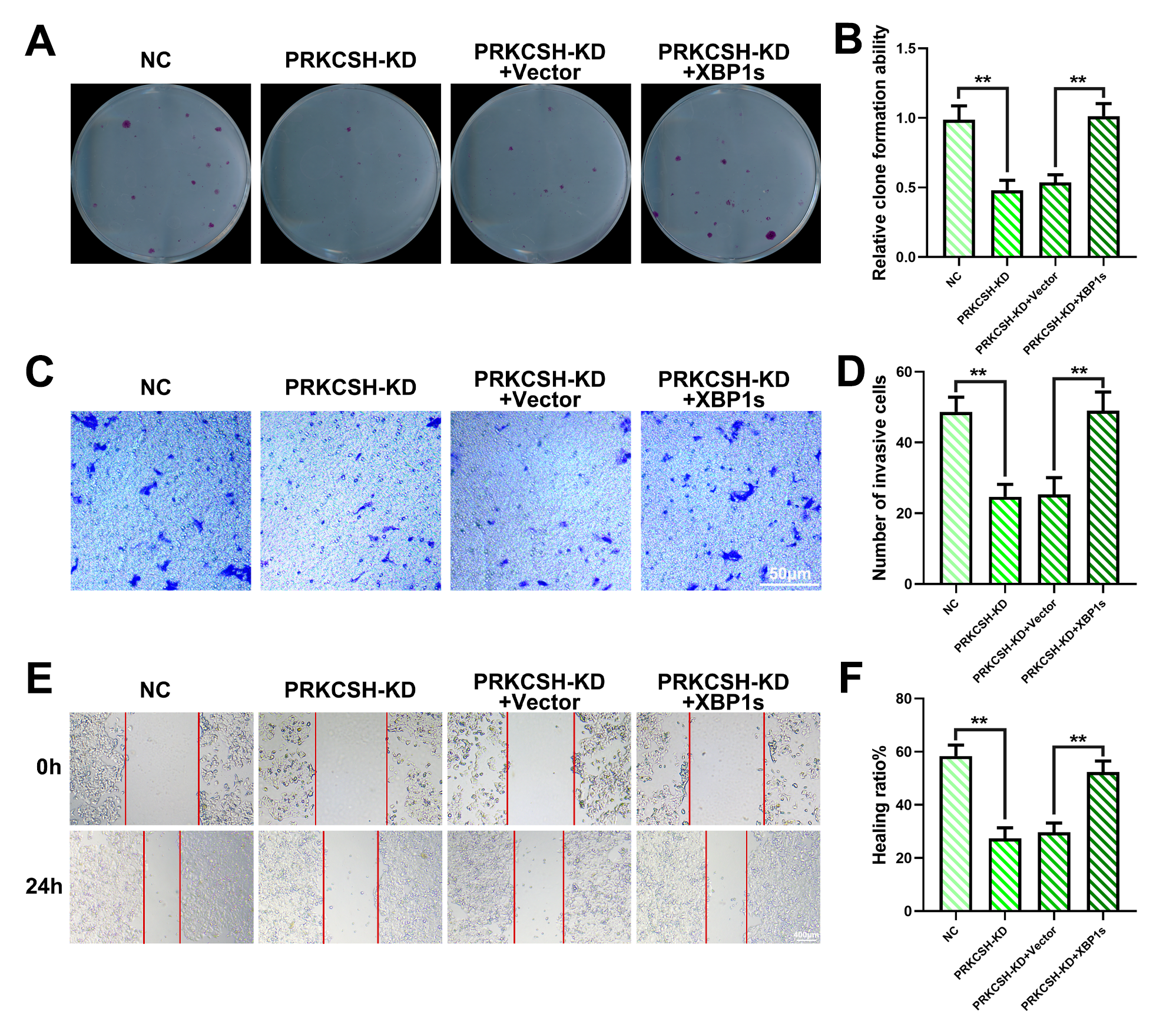

Supplement: Supplementary file 4 — Supplementary Figure 3 [file 41419_2025_7582_MOESM4_ESM.tif]

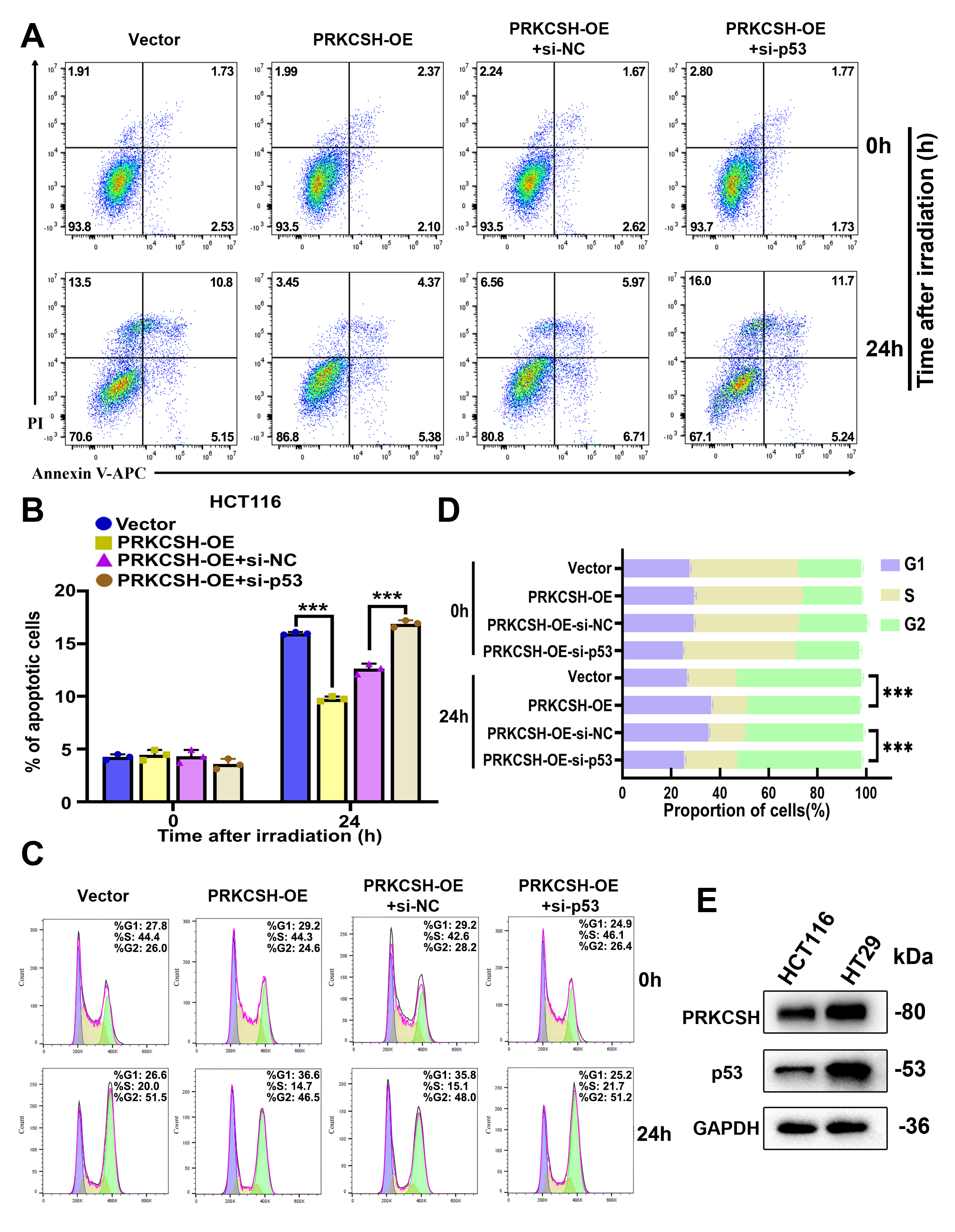

Supplement: Supplementary file 5 — Supplementary Figure 4 [file 41419_2025_7582_MOESM5_ESM.tif]

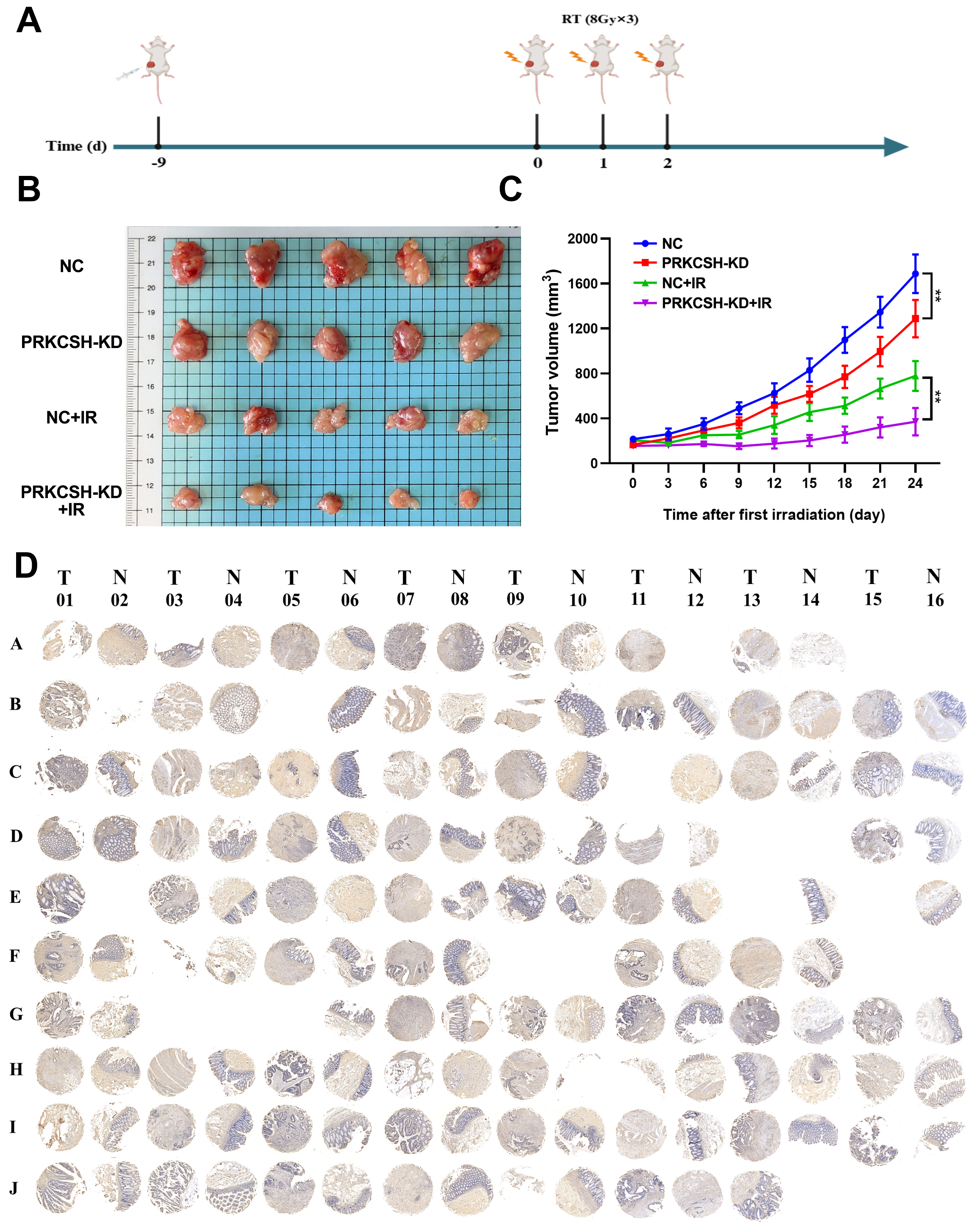

Supplement: Supplementary file 6 — Supplementary Figure 5 [file 41419_2025_7582_MOESM6_ESM.tif]
