# Supplementary material for: PRKCSH enhances colorectal cancer radioresistance via IRE1α/XBP1s-mediated DNA repair
Source: Cell Death Dis. 2025 Apr 6;16(1):258. doi: 10.1038/s41419-025-07582-4 (PMC11973196; doi:10.1038/s41419-025-07582-4)
Supplement: Supplementary file 7 — Full and uncropped western blots [file 41419_2025_7582_MOESM7_ESM.pdf]

Fig1.C

1C-HCT116-PRKCSH-IR

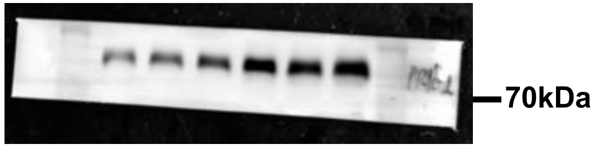

1C-HCT116-GAPDH-IR

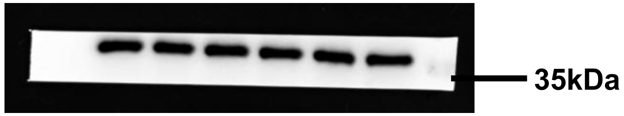

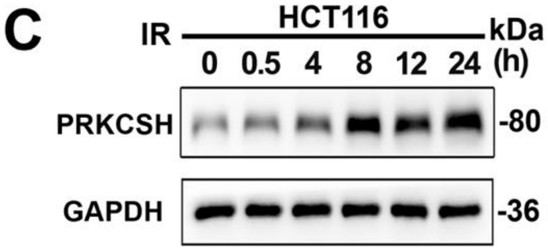

Fig1.D

1D-RKO-PRKCSH-IR

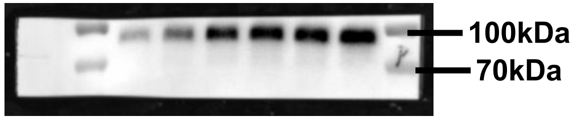

1D-RKO-GAPDH-IR

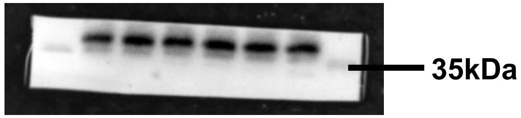

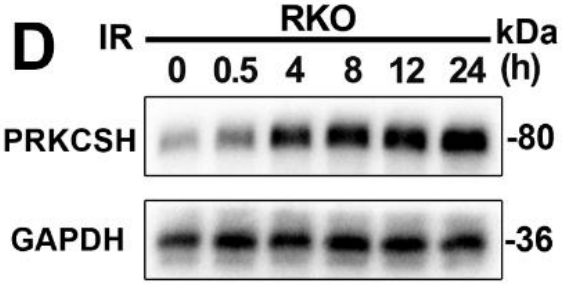

Fig1.E

1E-HCT116-PRKCSH-Knockdown

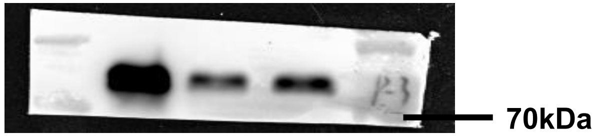

1E-HCT116-GAPDH-Knockdown

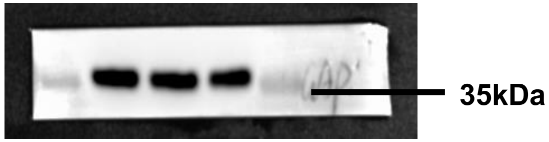

1E-RKO-PRKCSH-Knockdown

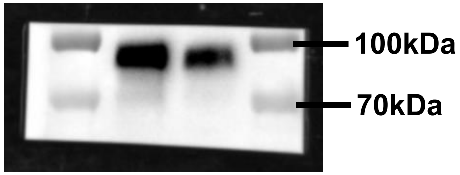

1E-RKO-GAPDH-Knockdown

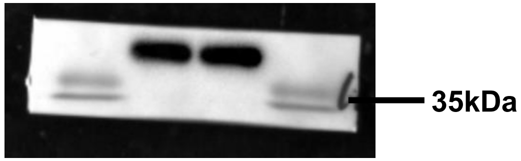

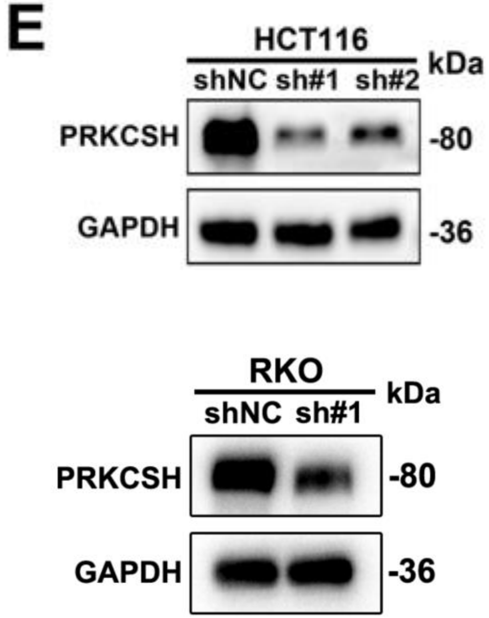

Fig2.G

2G-HCT116-PRKCSH-IR

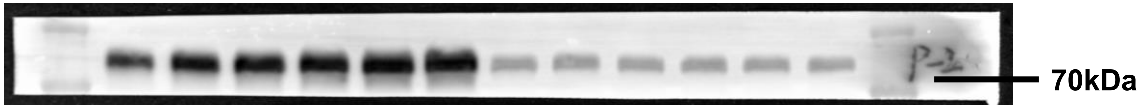

2G-HCT1116-C-Caspase3-IR

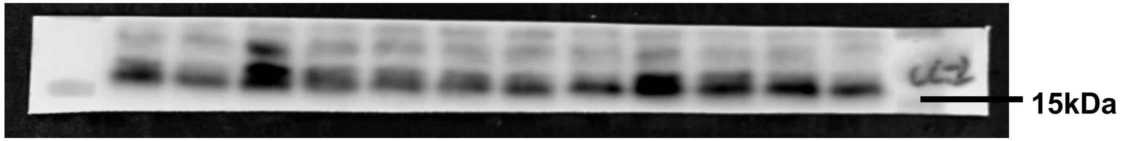

2G-HCT116-Bax-IR

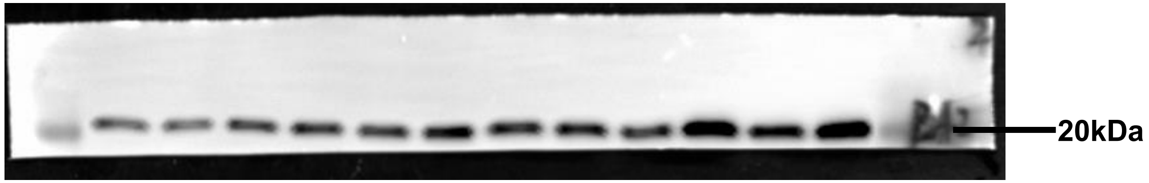

2G-HCT116-Bcl2-IR

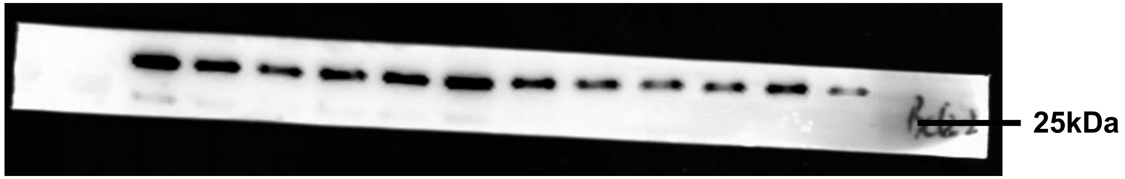

2G-HCT116-GAPDH-IR

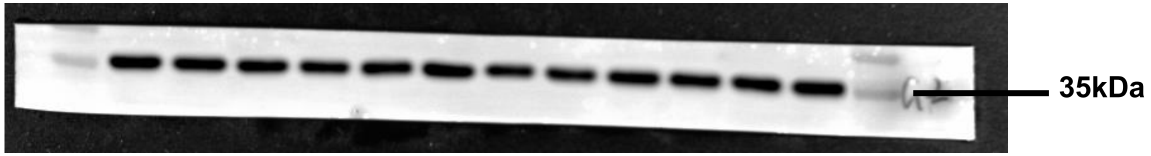

G

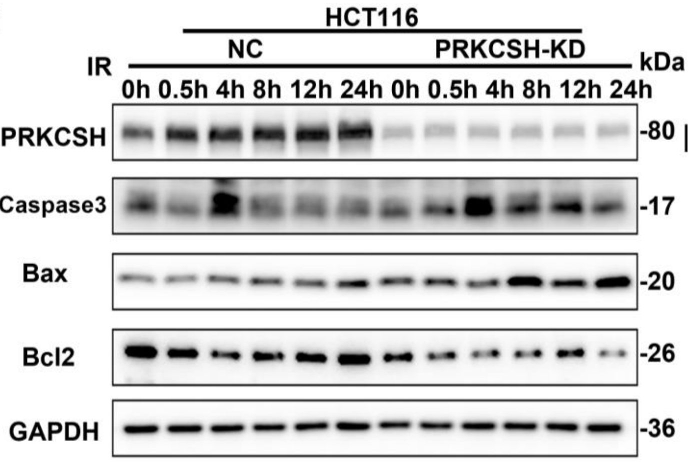

<table>
<tr><td>Fig3.A</td><td></td><td rowspan="7">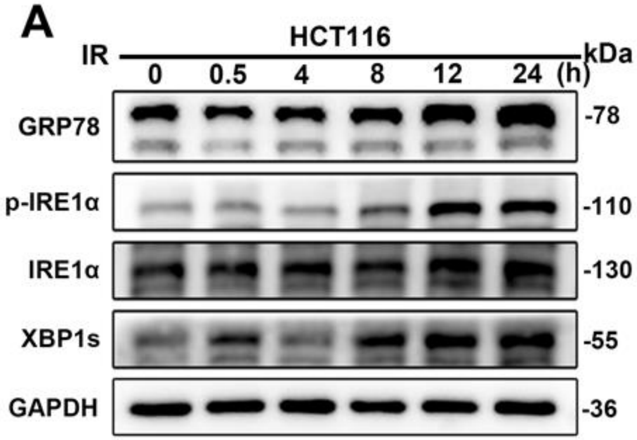
</td></tr>
<tr><td>3A-HCT116-GRP78-IR</td><td>3A-HCT116-p-IRE1α-IR</td></tr>
<tr><td>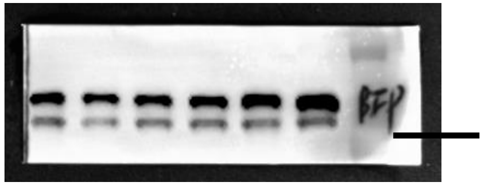 70kDa</td><td>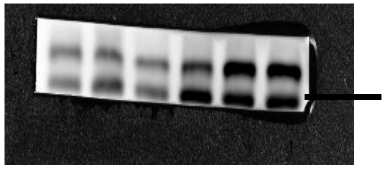 100kDa</td></tr>
<tr><td>3A-HCT116-IRE1α-IR</td><td>3A-HCT116-XBP1s-IR</td></tr>
<tr><td>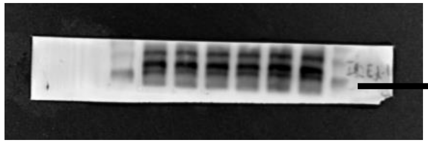 130kDa</td><td>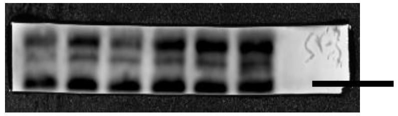 50kDa</td></tr>
<tr><td>3A-HCT116-GAPDH-IR</td><td></td></tr>
<tr><td>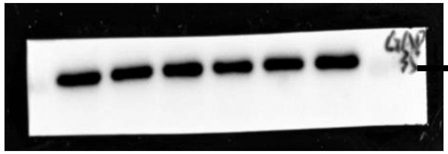 35KDa</td><td></td></tr>
</table>

Fig3.F

3F-HCT116-PRKCSH-IR

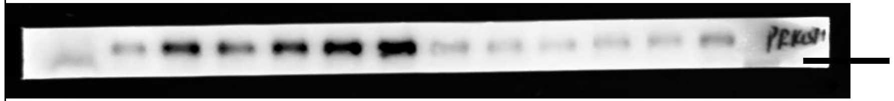

70kDa

3F-HCT116-GRP78-IR

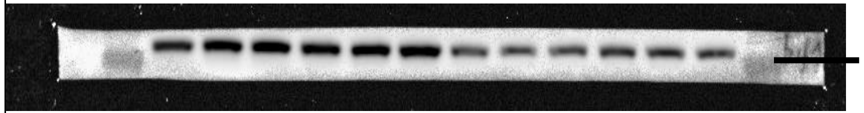

70kDa

3F-HCT116-p-IRE1α-IR

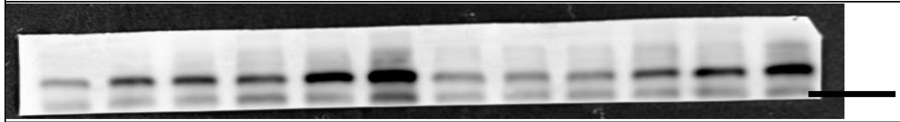

100kDa

3F-HCT116-IRE1α-IR

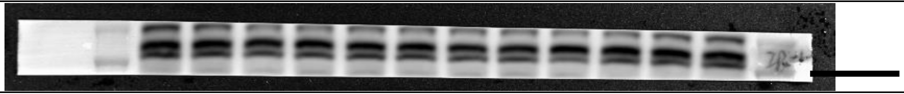

100kDa

3F-HCT116-XBP1s-IR

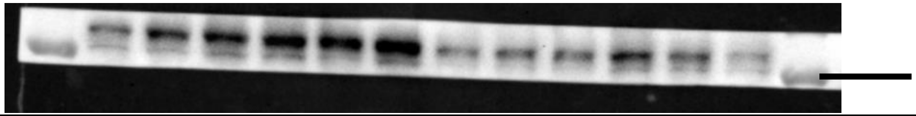

55kDa

3F-HCT116-PERK-IR

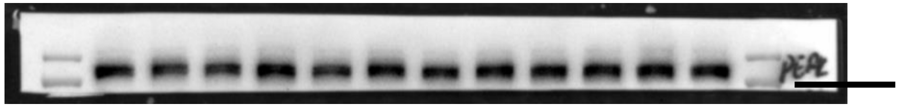

130kDa

3F-HCT116-ATF6-IR

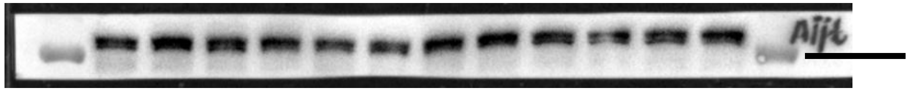

100kDa

3F-HCT116-GAPDH-IR

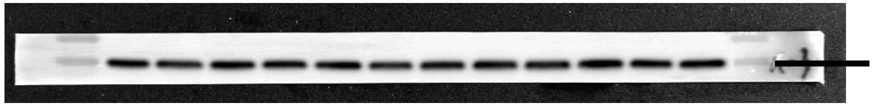

35kDa

F

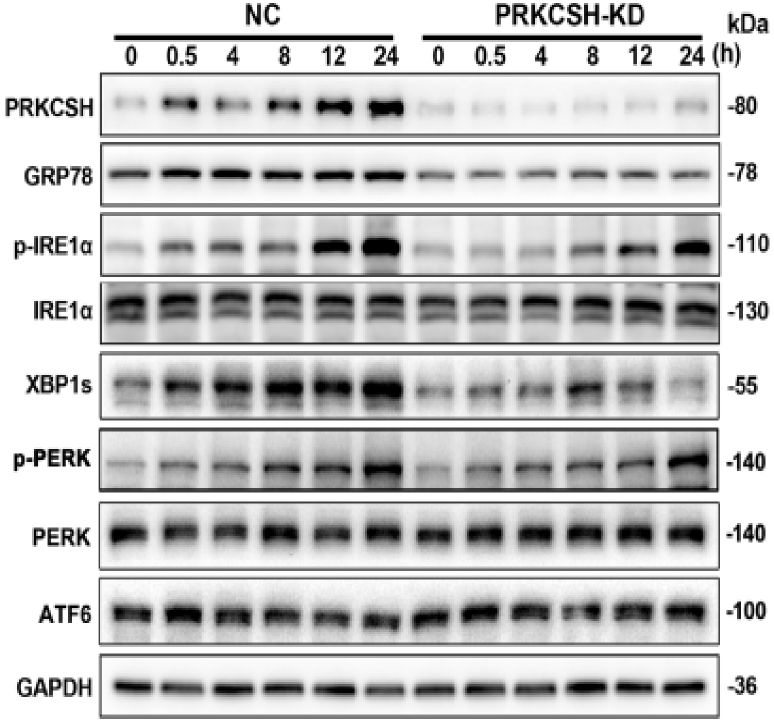

3F-HCT116-PERK-IR

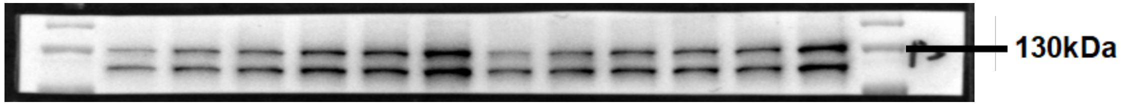

Fig3.G

3G-HCT116-p-IREIα

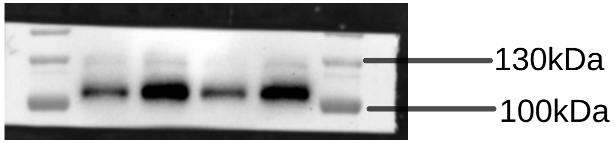

3G-HCT116-IREIα

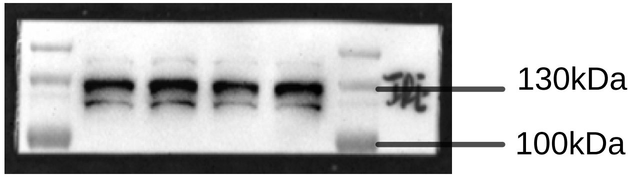

3G-HCT116-XBP1s

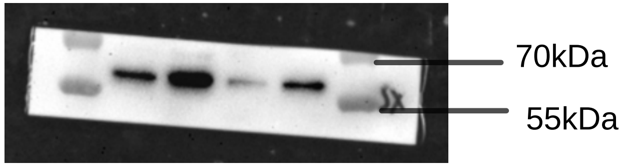

3G-HCT116-GAPDH

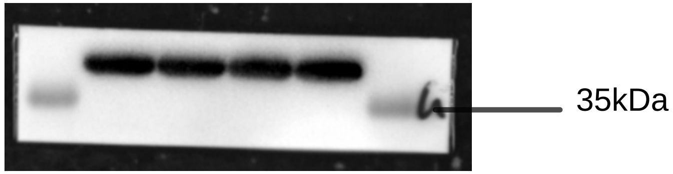

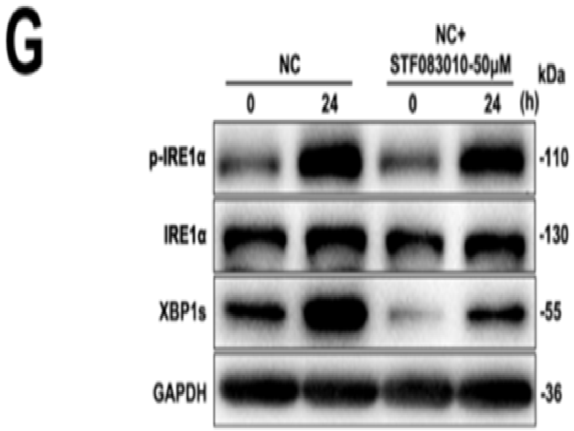

Fig4.G

4G-HCT116-PRKCSH-IR

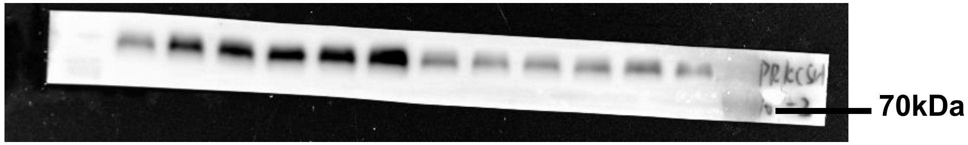

70kDa

4G-HCT116-γH2AX-IR

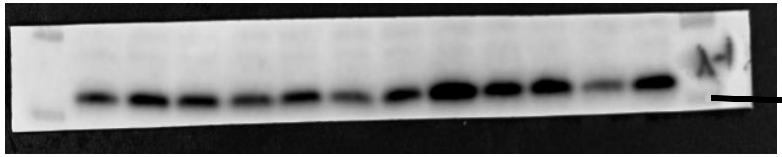

15kDa

4G-HCT116-BRCA1-IR

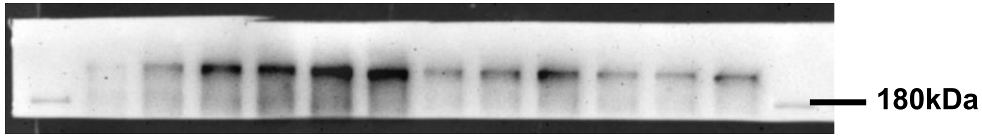

180kDa

4G-HCT116-p53-IR

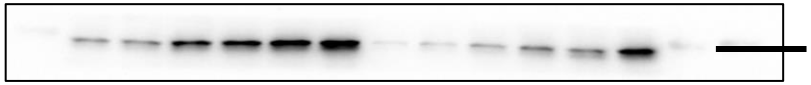

55kDa

4G-HCT116-p-RPA2-IR

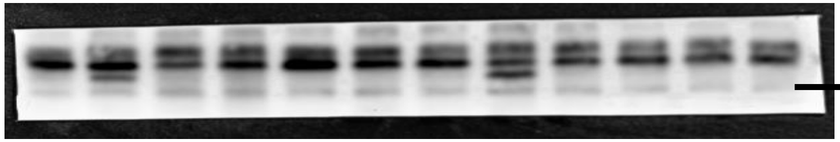

25kDa

4G-HCT116-p21-IR

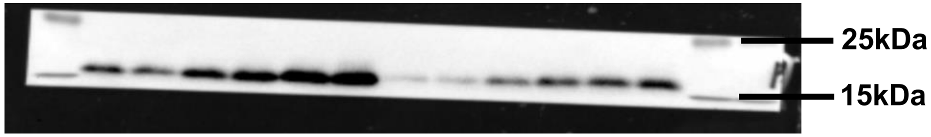

25kDa

15kDa

4G-HCT116-RPA2-IR

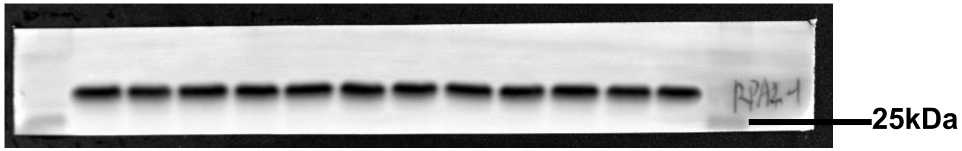

25kDa

4G-HCT116-p-CDC2-IR

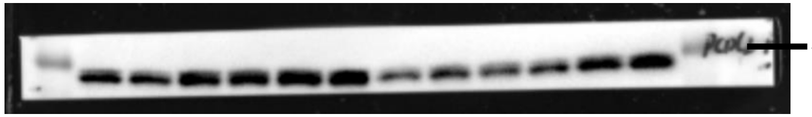

35kDa

4G-HCT116-RAD51-IR

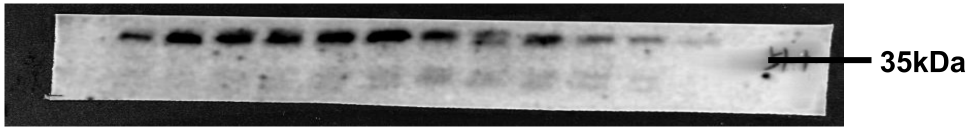

35kDa

4G-HCT116-CDC2-IR

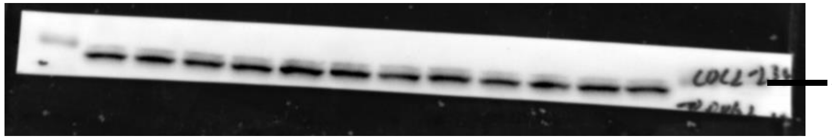

35kDa

Fig4.G

4G-HCT116-CyclinB1-IR

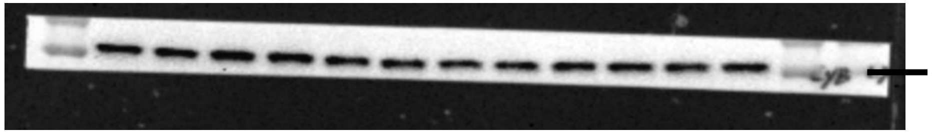

55kDa

4G-HCT116-GAPDH-IR

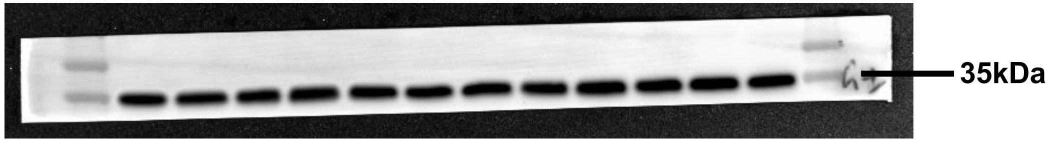

35kDa

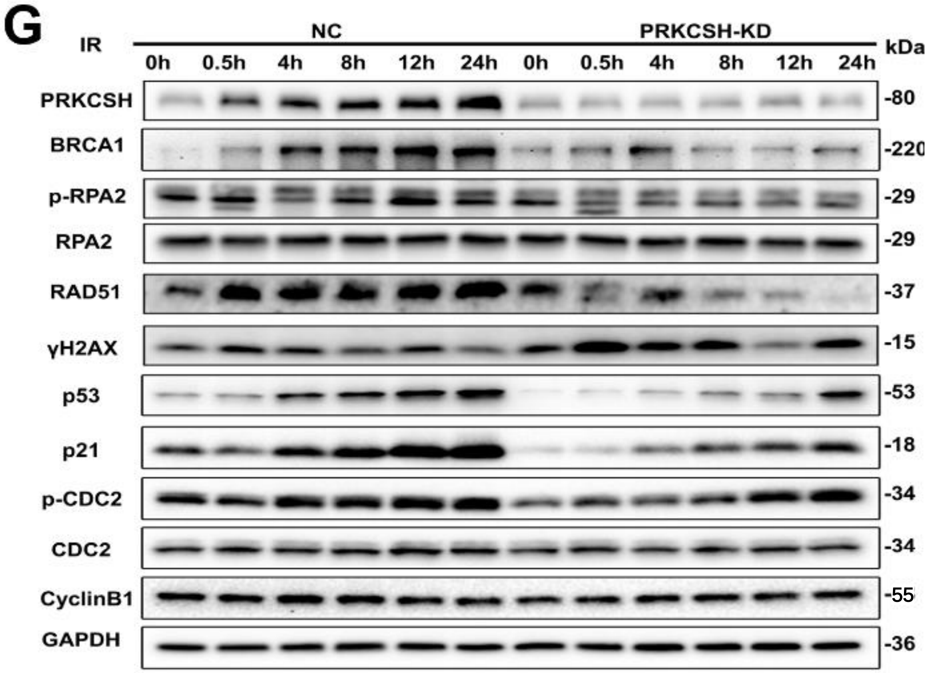

Fig5.B

5B-HCT116-PRKCSH-IR

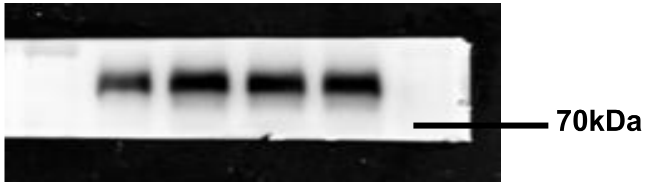

5B-HCT116-IRE1α-IR

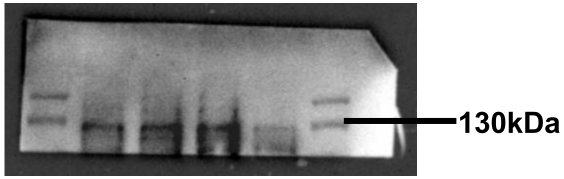

5B-HCT116-p-IRE1α-IR

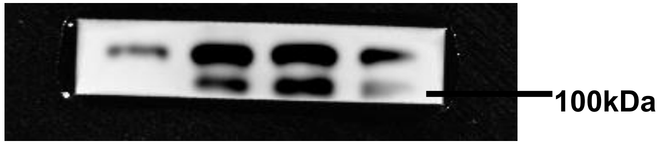

5B-HCT116-XBP1s-IR

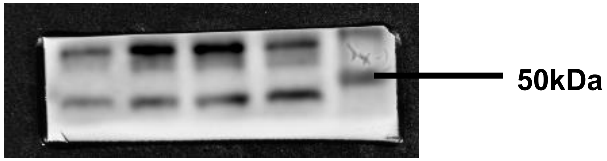

5B-HCT116- BRCA1-IR

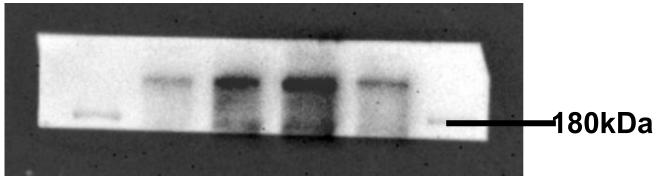

5B-HCT116-RAD51-IR

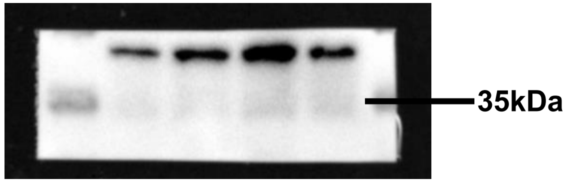

5B-HCT116-γH2AX-IR

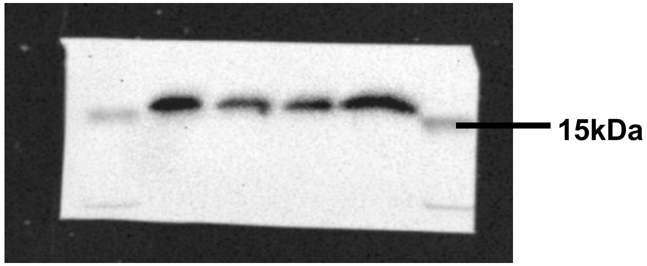

5B-HCT116- GAPDH -IR

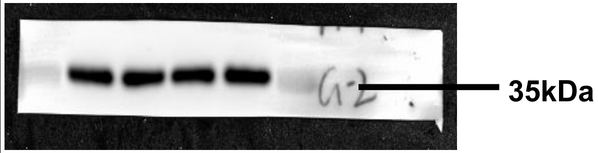

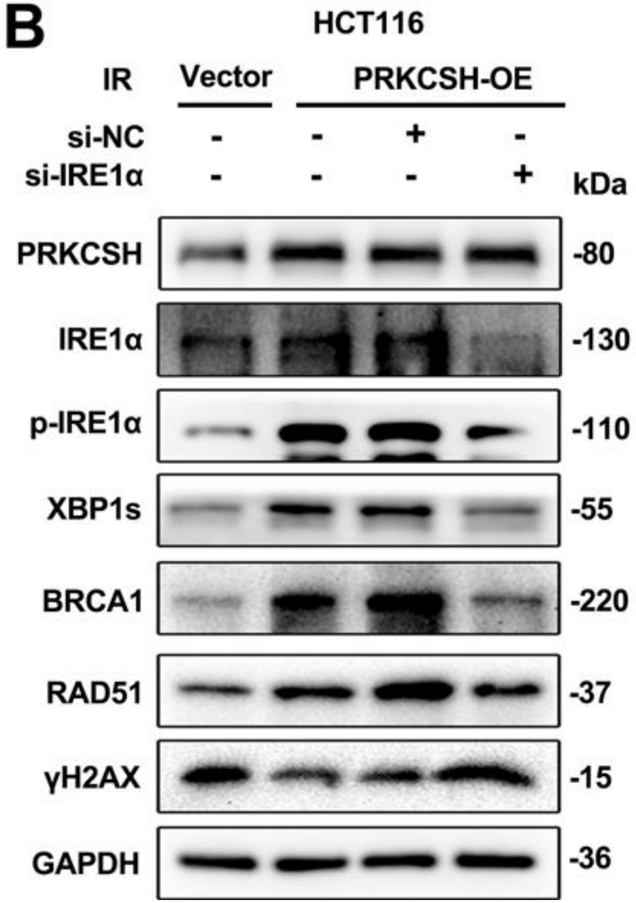

Fig6.B

6B-HCT116-PRKCSH

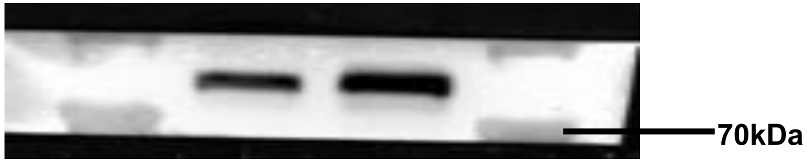

6B-HCT116-XBP1S

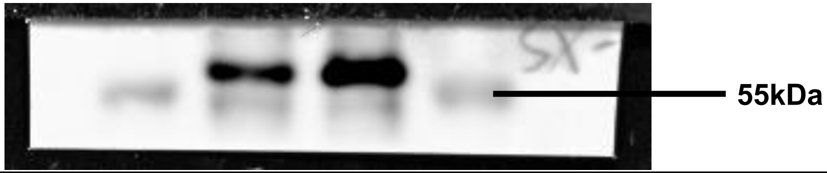

6B-HCT116-p53

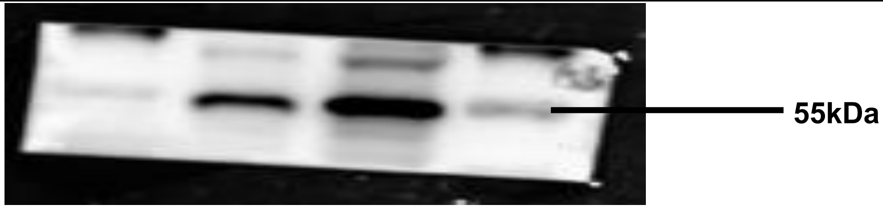

6B-HCT116-p21

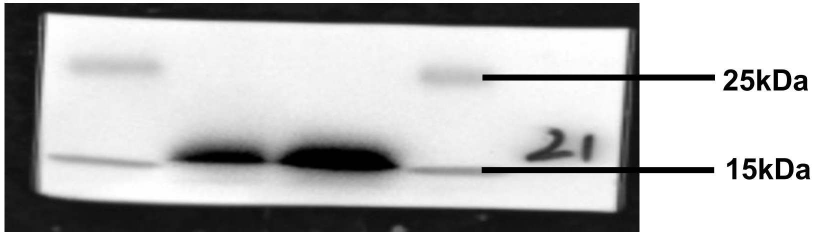

6B-HCT116-GAPDH

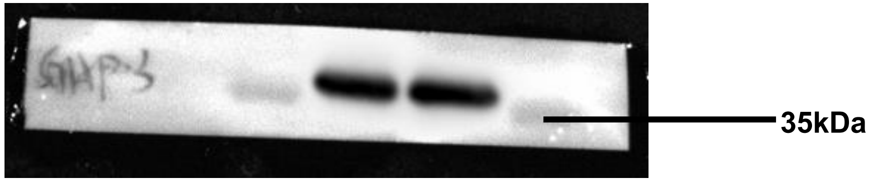

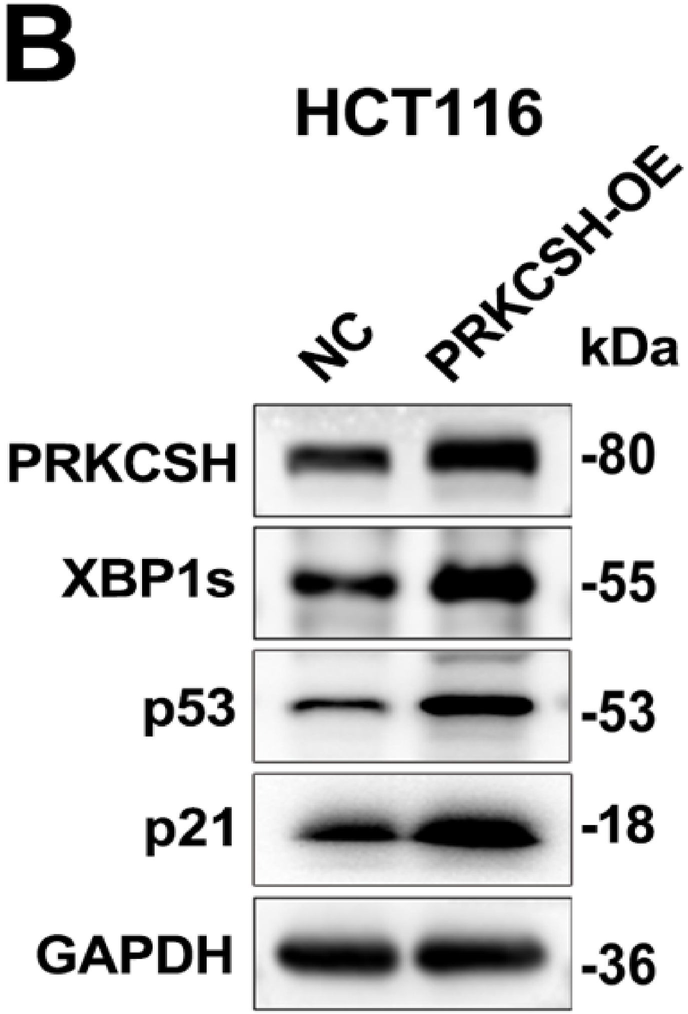

Fig6.C

6C-HCT116-PRKCSH-IR

Input **IP**

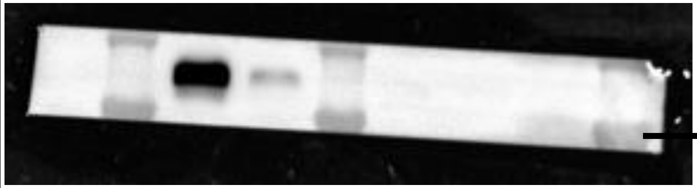

70kDa

6C-HCT116-XBP1s-IR

Input **IP**

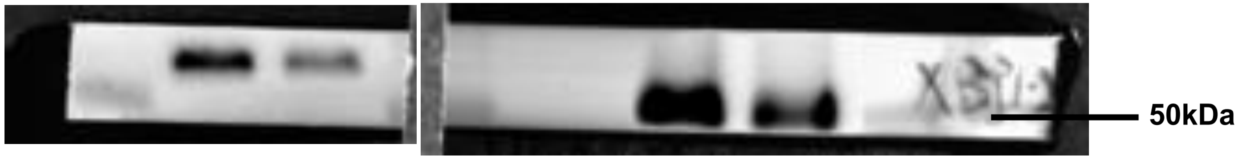

50kDa

6C-HCT116-p53-IR

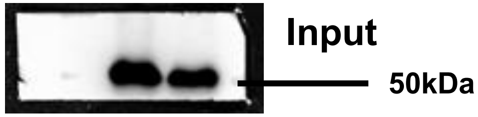

Input

50kDa

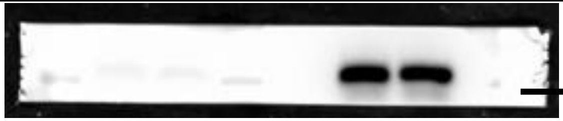

IP

50kDa

6C-HCT116-GAPDH-IR

Input **IP**

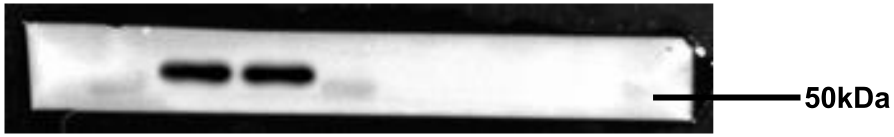

50kDa

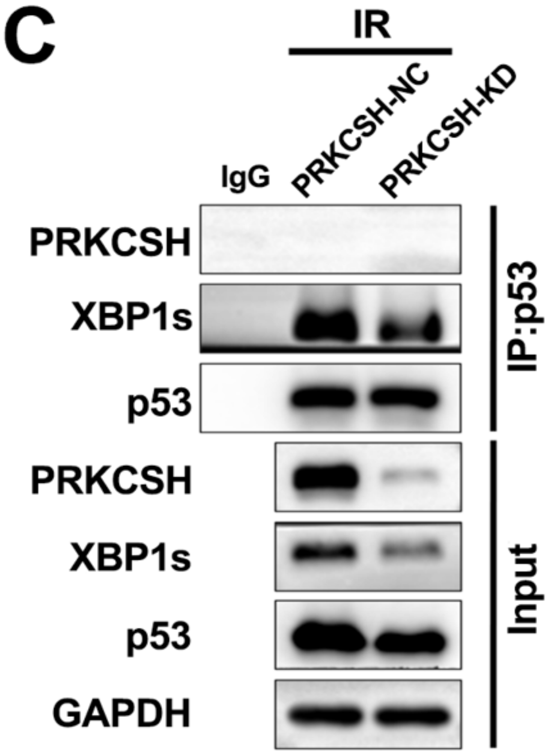

Fig6.D

6D-HCT116-PRKCSH-IR

Input IP

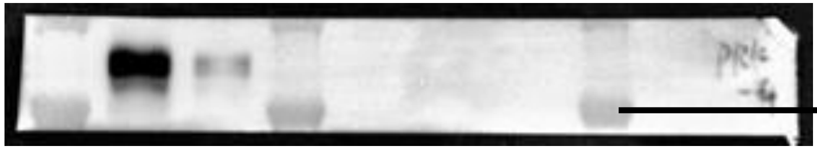

70kDa

6D-HCT116-p53-IR

Input IP IP

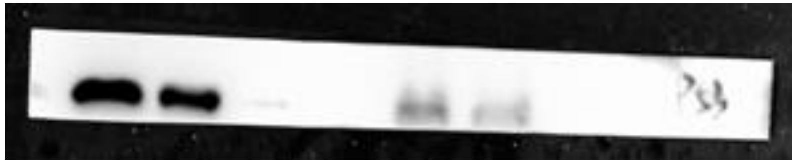

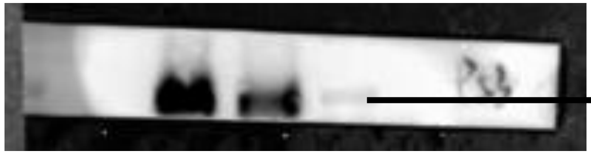

50kDa

6D-HCT116-XBP1s-IR

Input IP

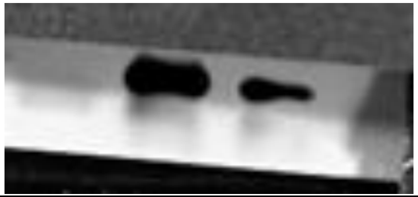

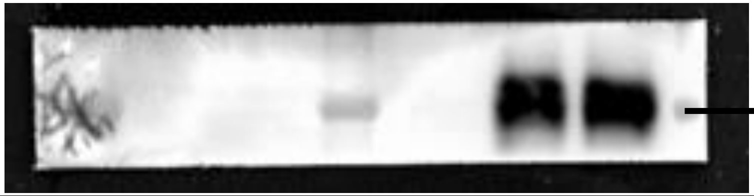

50kDa

6D-HCT116-GAPDH-IR

Input IP

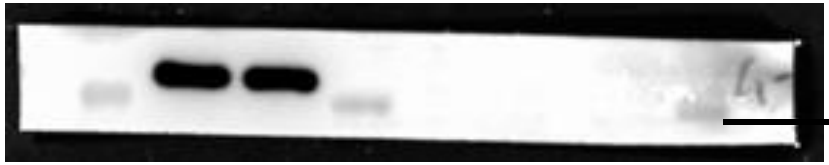

35kDa

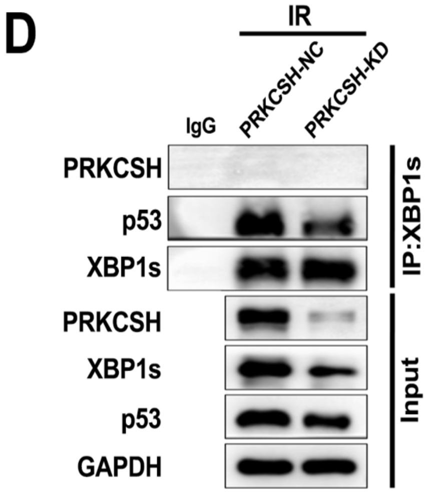

Fig6.F

6F-HCT116-P53-(ub)s-IP-IR

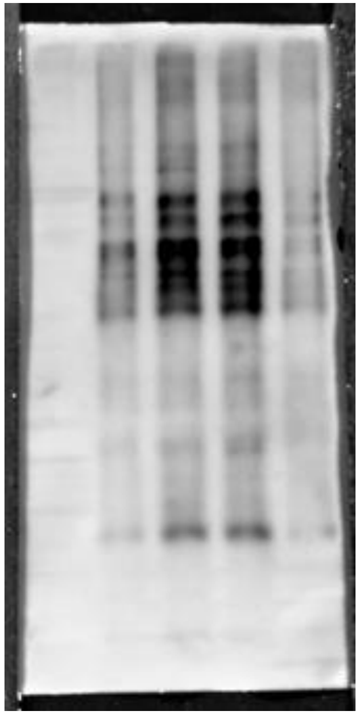

6F-HCT116-XBP1s-Input-IR

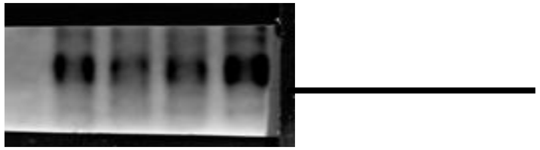

6F-HCT116-GAPDH-Input-IR

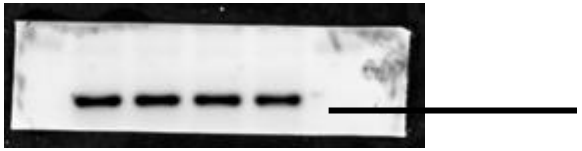

6F-HCT116-P53-IP-IR

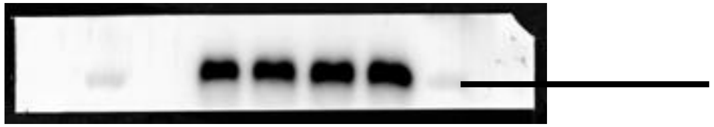

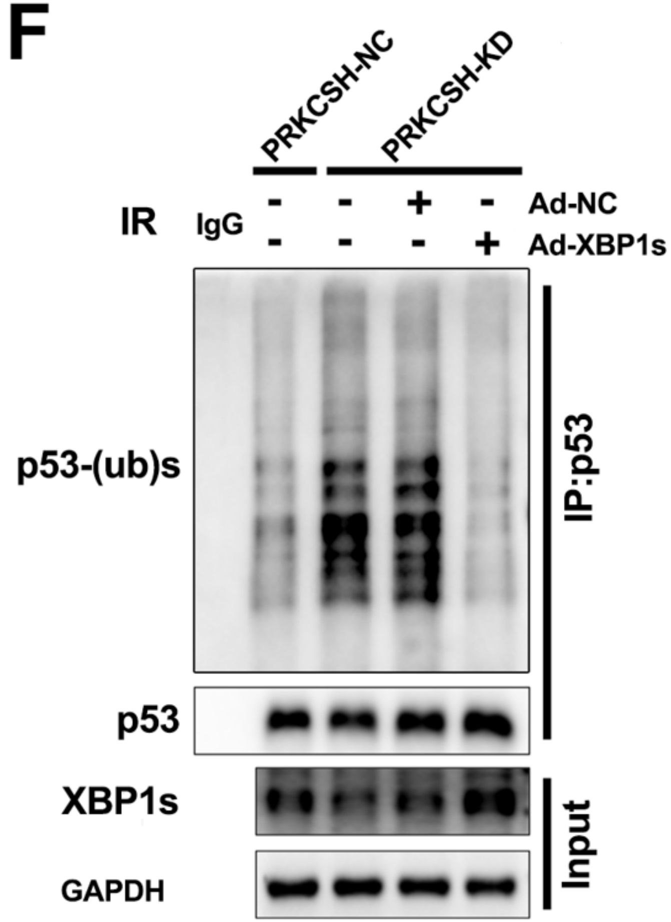

Fig7.E

7E-HCT116-PRKCSH-IR

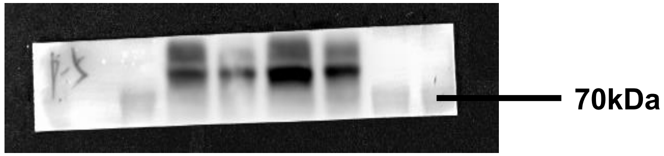

7E-HCT116-GRP78-IR

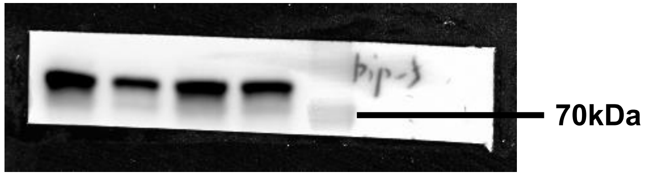

7E-HCT116-p-IRE1α-IR

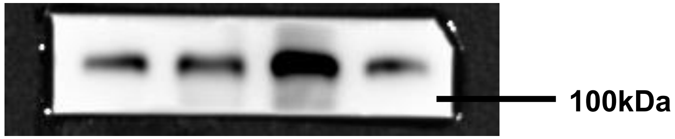

7E-HCT116-IRE1α-IR

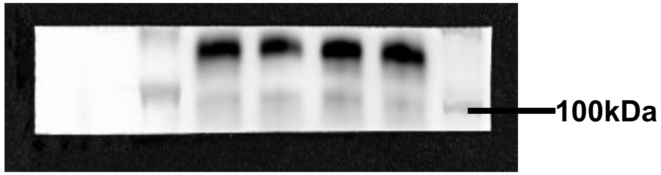

7E-HCT116-XBP1s-IR

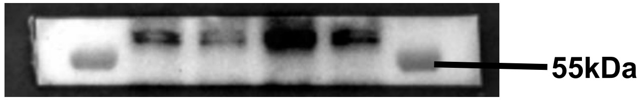

7E-HCT116-p53-IR

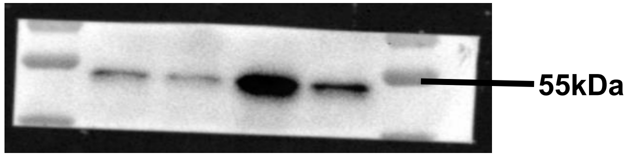

7E-HCT116-BRCA1-IR

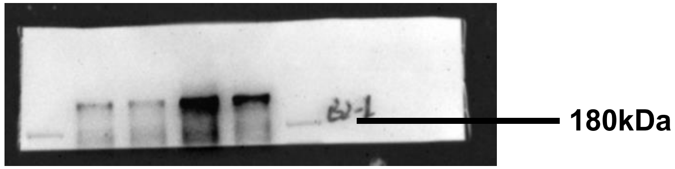

7E-HCT116-RAD51-IR

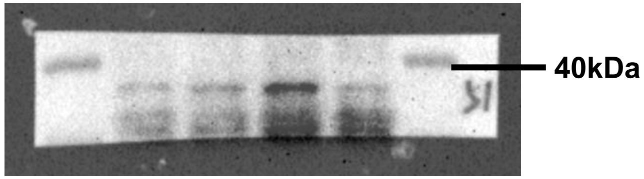

7E-HCT116-γH2AX-IR

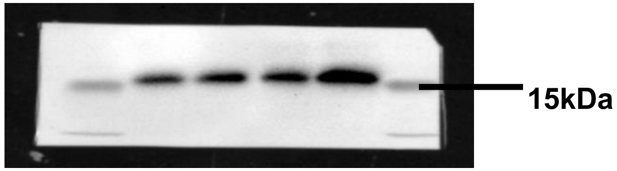

7E-HCT116-GAPDH-IR

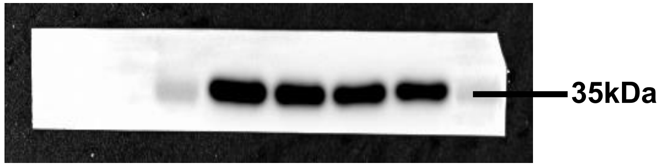

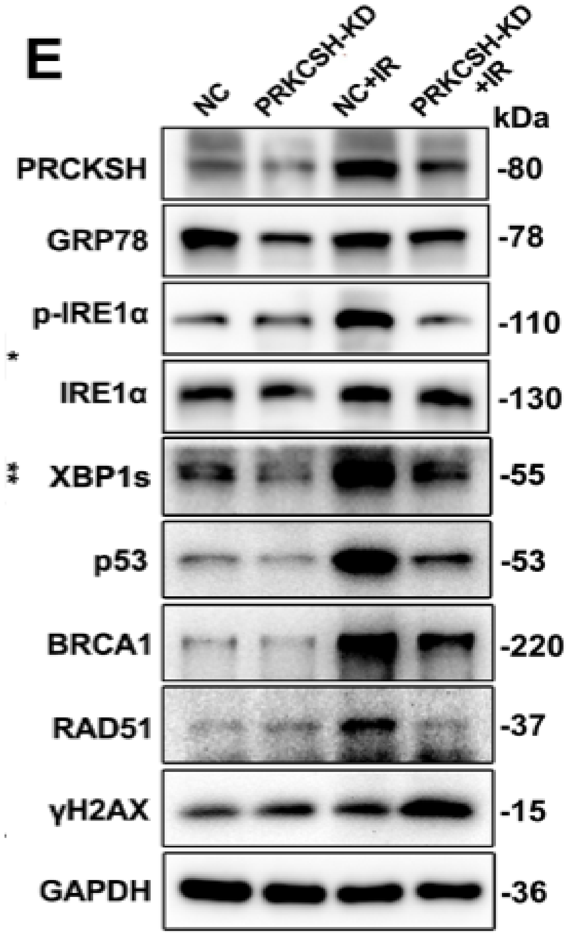

Fig8.C		
8C-PRKCSH-Knockdown	8C-GAPDH-Knockdown	
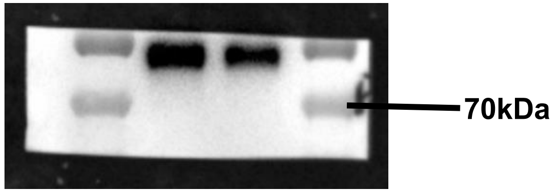	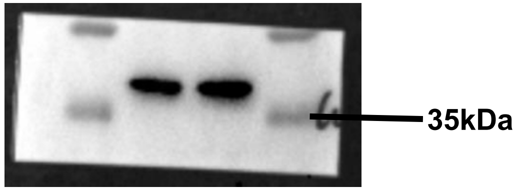	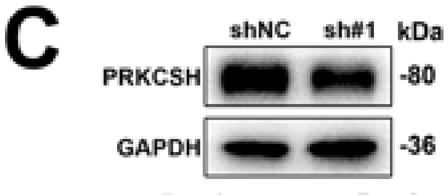

Fig Sup.1A		
Sup.1A-HT29-PRKCSH-IR	Sup.1A-HT29-GAPDH-IR	
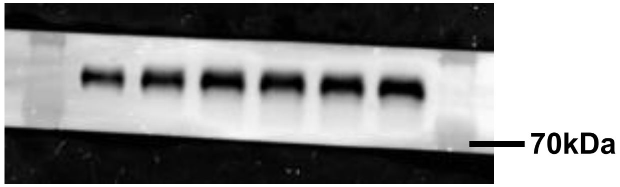	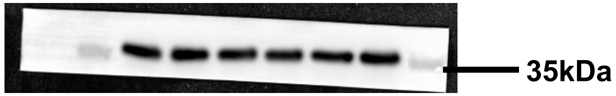	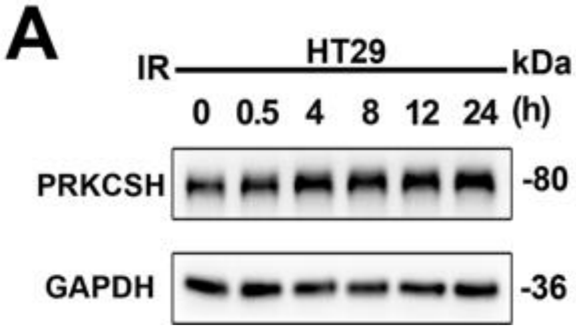

Fig Sup.1C		
Sup.1C-HT29-PRKCSH-Knockdown	Sup.1C-HT29-GAPDH-Knockdown	
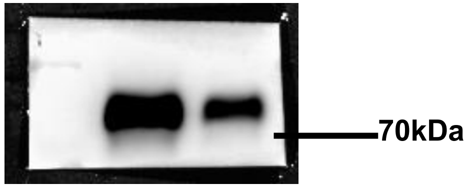	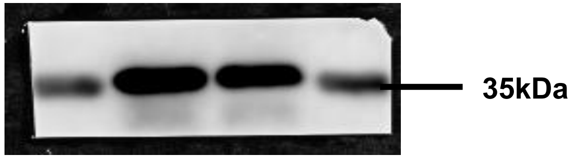	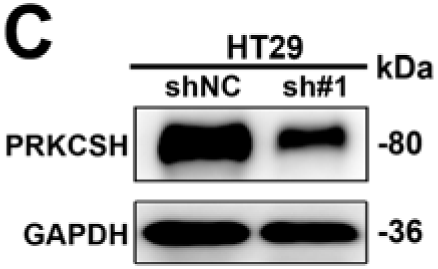

Fig Sup.2B		
Sup.2B-HCT116-PRKCSH	Sup.2B-HCT116-GAPDH	
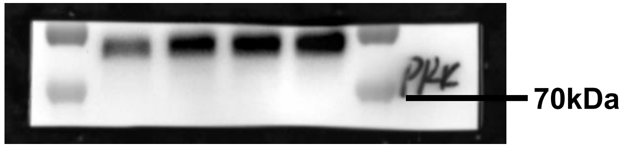	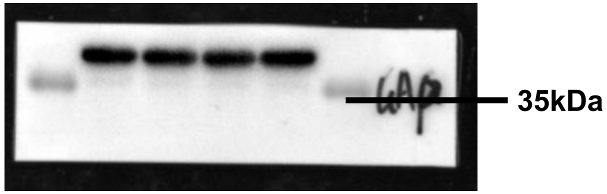	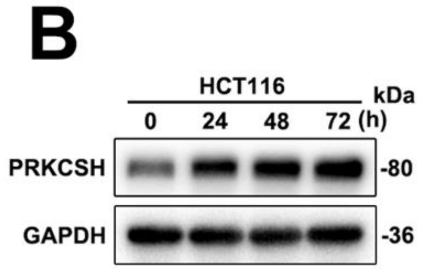

Fig Sup.2C		
Sup.2B-HCT116-PRKCSH-Overexpression	Sup.2C-HCT116-GAPDH-Overexpression	
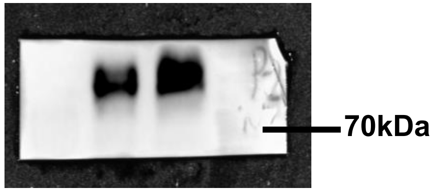	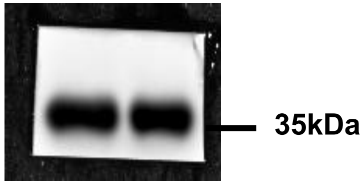	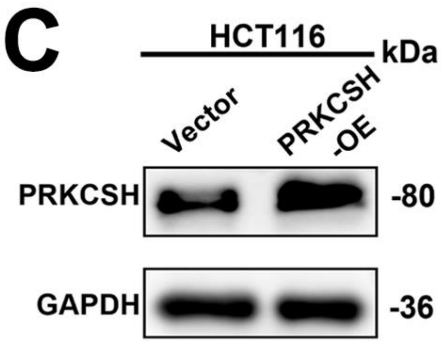

Fig Sup.2F

Sup.2F-HCT116-PRKCSH-IR

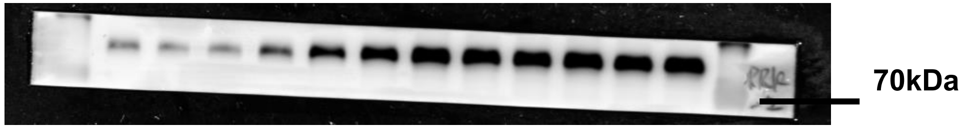

Sup.2F-HCT116-GRP78-IR

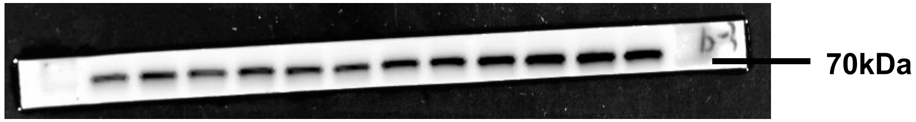

Sup.2F-HCT116-p-IRE1α-IR

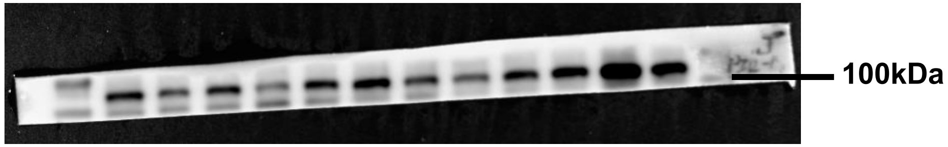

Sup.2F-HCT116-XBP1s-IR

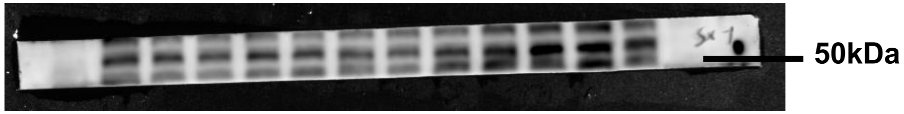

Sup.2F-HCT116-γH2AX-IR

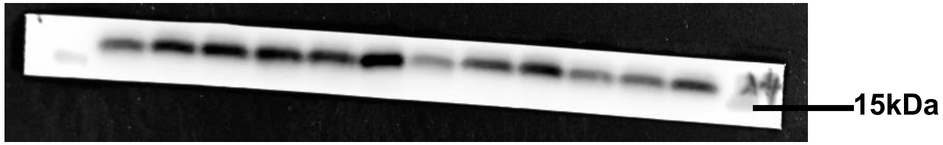

Sup.2F-HCT116-Bax-IR

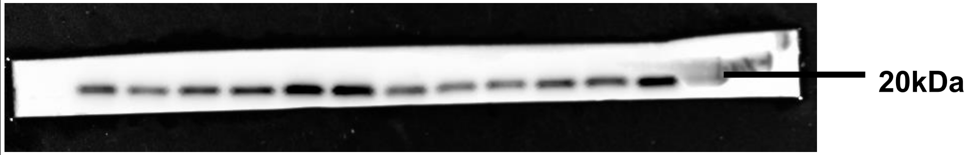

Sup.2F-HCT116-Bcl2-IR

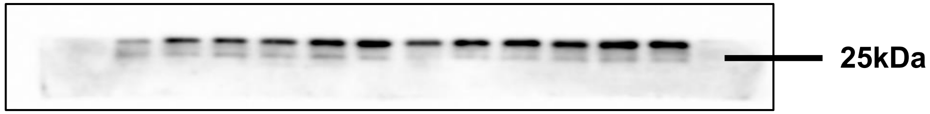

Sup.2F-HCT116-GAPDH-IR

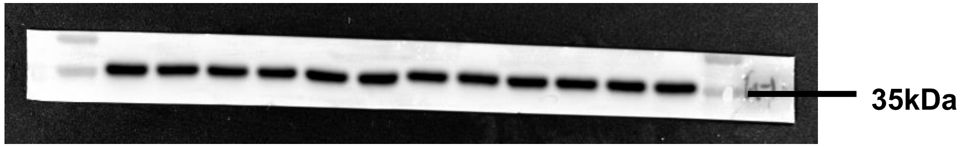

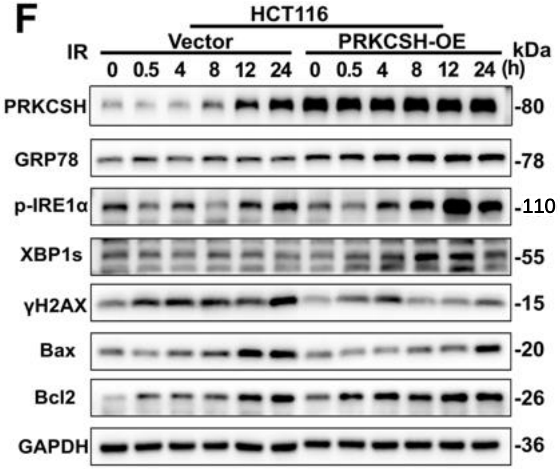

Fig Sup.4E

Sup.4E-PRKCSH

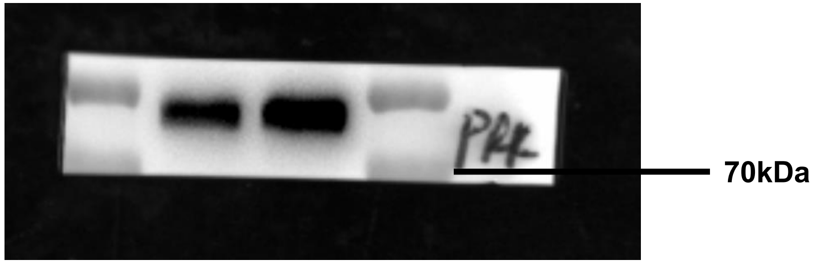

Sup.4E-p53

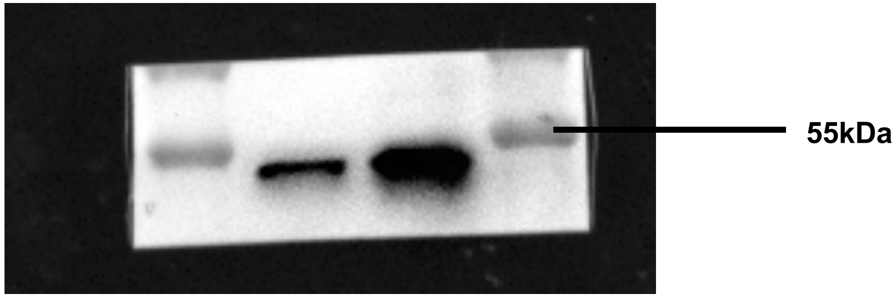

Sup.4E-GAPDH

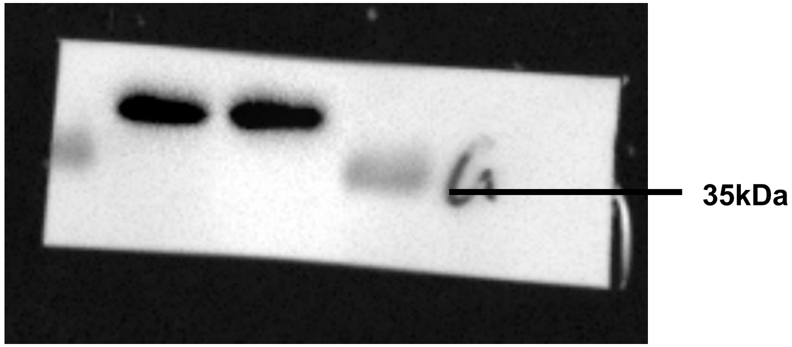

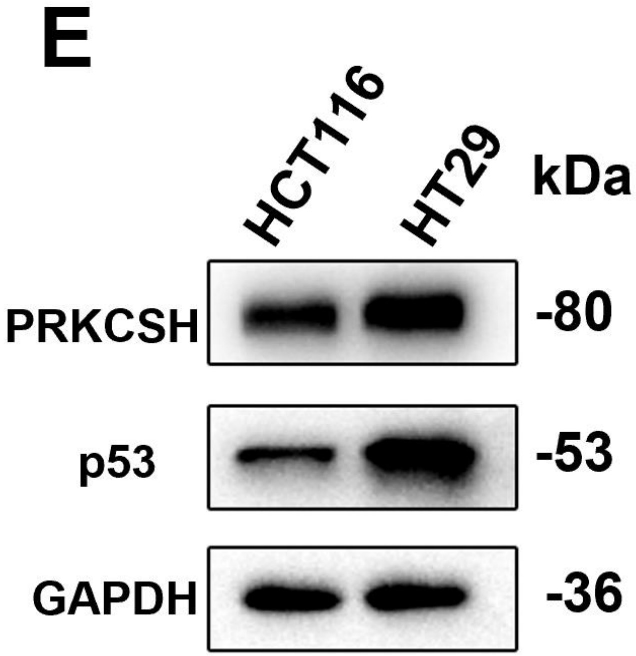

Marker Name	Catalog Number	Manufacturer	Country
Tricolor Prestained Protein Marker	WJ103	Epizyme Biotech	China
Prestained Protein Ladder	26616	Thermo Fisher Scientific	USA
